# One-pot synthesis of tetracyclic fused imidazo[1,2-*a*]pyridines via a three-component reaction

**DOI:** 10.3762/bjoc.12.145

**Published:** 2016-07-18

**Authors:** Bo Yang, Chuanye Tao, Taofeng Shao, Jianxian Gong, Chao Che

**Affiliations:** 1 Laboratory of Chemical Genomics, Engineering Laboratory for Chiral Drug Synthesis, School of Chemical Biology and Biotechnology, Peking University Shenzhen Graduate School, Shenzhen 518055, China

**Keywords:** Groebke–Blackburn–Bienaymé reaction, imidazo[1,2*-a*]pyridines, multi-component reaction, one-pot reaction

## Abstract

A novel three-component reaction has been developed to assemble biologically and pharmaceutically important tetracyclic fused imidazo[1,2*-a*]pyridines in a one-pot fashion utilizing readily available 2-aminopyridines, isatins and isocyanides. The three-component coupling proceeds through the Groebke–Blackburn–Bienaymé reaction followed by a retro-aza-ene reaction and subsequent nucleophilic reaction of the in-situ generated imidazo[1,2*-a*]pyridines bearing an isocyanate functional group.

## Introduction

Multicomponent reactions (MCRs) have attracted considerable attention in organic and medicinal chemistry due to their high efficiency, simple operability, atom economy and unmatched versatility [1–6]. Especially, these reactions serve as an ideal synthetic tool for the assembly of structurally diverse and biologically relevant heterocycles, and thus have been extensively investigated by organic and medicinal chemists to explore lead compounds in drug discovery efforts [7–10].

The imidazo[1,2*-a*]pyridine scaffold is a pharmaceutically important drug template, and its derivatives display a broad range of biological activities such as antibacterial [11–13], antiviral [14–15], anti-inflammatory [16–17], antitumor [18–20], and anti-HIV [21]. It is found as the core structure in several drugs such as Zolpidem, Alpidem and Zolimidine (approved for treatments of insomnia, anxiety and peptic ulcers, respectively) [22]. As such, the imidazo[1,2*-a*]pyridine structure represents an intriguing synthetic target, and its further functionalization is leading to polycyclic fused heterocycles that may have interesting biological profiles [23].

Impressively, the imidazo[1,2*-a*]pyridine scaffolds can be constructed in great diversity by a multicomponent reaction of amidines, aldehydes and isocyanides. This MCR, a variant of the Ugi reaction [24–25], was discovered independently by three groups and is known as the Groebke–Blackburn–Bienaymé (GBB) reaction [26–29]. The reaction involves a formal [4 + 1] cycloaddition of isocyanides [30] and imines, generated from the amidines and aldehydes, allowing straightforward access to diverse imidazo[1,2-*x*]azines [31–33].

**Scheme 1:**
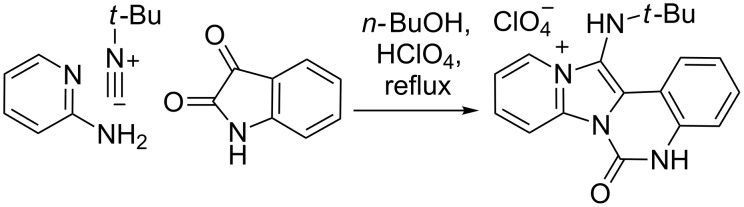
MCR to polycyclic fused imidazo[1,2-*a*]pyridine derivatives.

In view of the significance of the GBB reaction and the imidazo[1,2*-a*]pyridine core structure, the further development of new GBB-based methods for the efficient synthesis of novel polycyclic fused imidazo[1,2*-a*]pyridines is highly desirable. In an earlier study, we have developed a GBB/lactamization MCR strategy, which provided the rapid access to isoquinolinone-fused imidazo[1,2*-a*]pyridines with potent and selective CDK2 inhibition properties [34–35]. As a continuing effort, we report herein our recent efforts in the development of a GBB-based MCR method for the one-pot synthesis of diverse quinazolin-2-one fused imidazo[1,2*-a*]pyridines (Scheme 1). Parts of the work have been disclosed in a previous patent [36].

## Results and Discussion

It is noteworthy that there was no report on a ketone-involving GBB reaction, and several attempts to explore the GBB reaction utilizing ketones as carbonyl reactant failed [27,37]. These difficulties could be partially explained in terms of the electronic and steric effect of the ketone involved. More importantly, when a ketone is used in the reaction the formed unstable [4 + 1] cycloaddition adduct could not undergo a [1,3]-alkyl shift. On the other hand the reaction with an aldehyde allows further conversion through a [1,3]-hydride shift to form a stable and aromatic imidazole. Therefore, we envisioned that a ketone-involved reaction could proceed, if the [4 + 1] cycloaddition adduct can further rearrange to form an aromatic imidazole. With this idea in mind, we started to study an isatin-involved GBB reaction [38–39], because the resulting [4 + 1] cycloaddition adduct could further proceed with a retro-aza–ene reaction via a concerted [1,5]-hydride shift.

We first explored the reaction of 2-aminopyridine (**2a**), isatin (**3a**) and *tert*-butyl isocyanide (**4a**). Under the classical reaction conditions (entry 1, Table 1), the product **1a** was isolated only in 4% yield. Performing the reaction under reflux conditions slightly improved the yield to 11%. We next examined solvent effects in this MCR reaction and various solvents were screened, as summarized in Table 1.

**Table 1:** Synthesis of imidazo[1,2-*a*]pyridine derivatives in indicated conditions [36].^a^

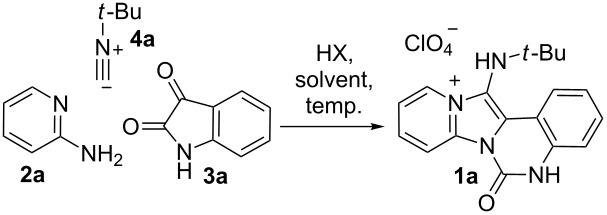				

Entry	Solvent	Acid	Temperature	Yield (%)

1	MeOH	HClO_4_	rt	4
2	MeOH	HClO_4_	reflux	11
3	MeCN	HClO_4_	reflux	–^b^
4	DMF	HClO_4_	100 °C	10
5	EtOH	HClO_4_	reflux	17
6	iPrOH	HClO_4_	reflux	22
7	*n*-BuOH	HClO_4_	reflux	30
8	iBuOH	HClO_4_	reflux	21
9	*t*-BuOH	HClO_4_	reflux	19
10	isopentyl alcohol	HClO_4_	reflux	17
11	CF_3_CH_2_OH	HClO_4_	reflux	13
12	*n*-BuOH	PTSA	reflux	20
13	*n*-BuOH	HCl	reflux	–
14	*n*-BuOH	AcOH	reflux	–
**15**	***n*-BuOH**	**HClO_4_**	**reflux**	**42**^c^

^a^Conditions: **2a** (1 mmol), **3a** (1 mmol), **4a** (1mmol), and acid **HX** (1 mmol) in 4 mL of solvent; ^b^"–" indicates that the product was not obtained; ^c^conditions: **2a** (1.35 mmol), **3a** (1 mmol), **4a** (1.35 mmol), and acid **HX** (1 mmol) in 4 mL of solvent.

We observed that protic solvents with medium polarity can facilitate the reaction by product precipitation from the reaction mixture and *n*-BuOH proved to be the most suitable solvent in the reaction. Performing the reaction in refluxing *n*-BuOH in the presence of one equivalent of HClO_4_ for 8 h, compound **1a** was obtained in 30% isolated yield. Other acids including *p*-toluenesulfonic acid (PTSA), HCl, and HOAc were also screened, and the results indicated that the use of PTSA led to a slightly decreased yield, and weaker acids failed to promote this process. It is worth noting that the reaction proceeded incompletely and significant amounts of isatin (**3a**) were recovered. When increased amounts (1.35 equiv) of 2-aminopyridine (**2a**) and *tert*-butyl isocyanide (**4a**) were used, the yield of **1a** could be further improved to 42%.

**Figure 1:**
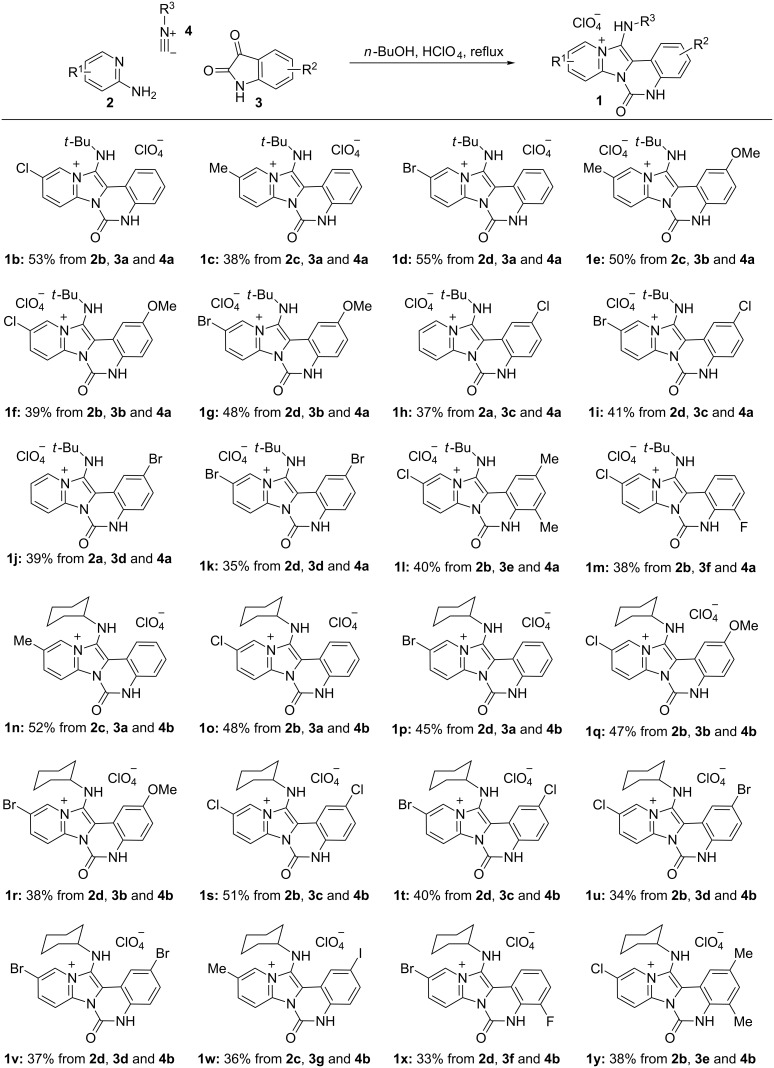
Syntheses of imidazo[1,2-*a*]pyridine derivatives. Reaction conditions: **2** (1.35 mmol), **3** (1 mmol), **4** (1.35 mmol), HClO_4_ (1 mmol), *n*-BuOH (4 mL), reflux. Yields refer to isolated yields. **2b** R^1^ = 4-Cl; **2c** R^1^ = 4-Me, **2d** R^1^ = 4-Br; **3b** R^2^ = 5-OMe; **3c** R^2^ = 5-Cl; **3d** R^2^ = 5-Br; **3e** R^2^ = 5,7-Me_2_; **3f** R^2^ = 7-F; **3g** R^2^ = 5-I; **4b** R^3^ = cyclohexyl.

Under the optimized reaction conditions, we started to investigate the reaction scope. The MCR of various 2-aminopyridines, isatins and isocyanides proceeded well under the optimized conditions and the combinational synthesis delivered structurally diverse quinazolin-2-one-fused imidazo[1,2*-a*]pyridines (Figure 1). It was found that the electronic properties of the substituents attached to the isatin and 2-aminopyridine had no obvious effect on the reactivity. When *tert*-butyl isocyanide (**4a**) was used, the reaction proceeded equally well with electron-withdrawing or electron-donating substituted isatins and 2-aminopyridines, delivering the desired products **1b**–**m** in 35–55% yield. Similar results were obtained from the reaction with cyclohexyl isocyanide (**4b**) to give **1n**–**y**. All products were characterized by ^1^H NMR, ^13^C NMR, HRMS spectra and the structure of **1o** was further confirmed by X-ray diffraction analysis (see Supporting Information File 1 for details).

**Figure 2:**
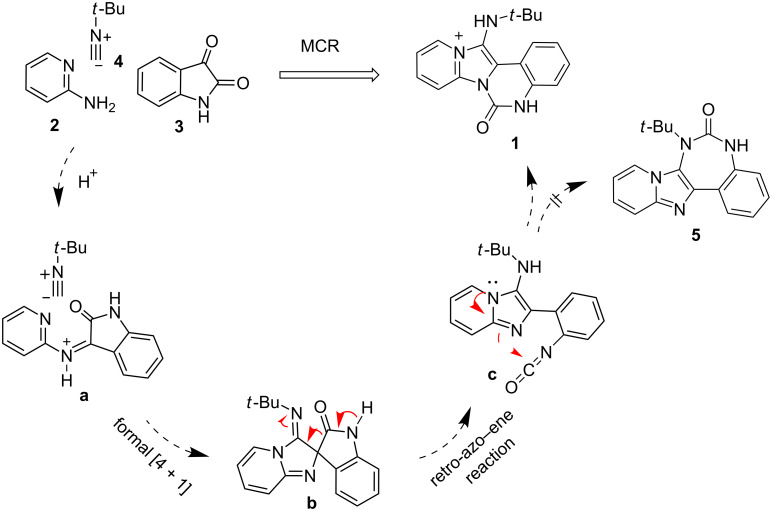
Mechanistic rationale for the MCR [36].

A possible mechanism for the MCR is proposed in Figure 2. The MCR proceeds through the formation of a protonated imine species from 2-aminopyridine and isatin which then undergoes a formal [4 + 1] cycloaddition with isocyanide to generate a spiro intermediate **b**. The spiro compound then undergoes a retro-aza–ene reaction via a [1,5]-hydride shift resulting in an aromatic imidazo[1,2*-a*]pyridine having an isocyanate functional group. A further intramolecular nucleophilic reaction of the imidazole and the newly generated isocyanate provides the final product **1**. Noteworthy, the benzodiazepinone-fused imidazo[1,2*-a*]pyridine **5** was not observed which may be due to the strain of the seven-membered ring.

## Conclusion

In conclusion we have developed a GBB-based multicomponent reaction of isatins, 2-animopyridines and isocyanides, which provides a direct and rapid access to diverse tetracylic fused imidazo[1,2*-a*]pyridines with moderate yields. To the best of our knowledge, the described chemistry represents the first example of the GBB reaction involving ketones, transforming the simple starting materials into complex heterocycles of pharmaceutical relevance in a highly efficient fashion. The developed MCR proceeds through a domino GBB/retro-aza–ene/nucleophilic cyclization process under mild reaction conditions, and its high efficiency and simple operation will make it have potential applications in the compound library synthesis.

## Experimental

**Typical procedure for multicomponent reaction.** To a solution of isatin (**3**, 1.0 mmol), 2-aminopyridine (**2**, 1.35 mmol) and isocyanide (**4**, 1.35 mmol) in 4 mL of *n*-butyl alcohol was added HClO_4_ (1.0 mmol), and the reaction mixture was stirred under refluxing temperature for 8 h. When the reaction mixture had cooled to room temperature, the formed precipitate was collected by filtration, rinsed with ethanol and dried to afford the target compounds **1**.

## Supporting Information

File 1 Experimental procedures, characterization and spectral data for synthesized compounds and X-ray data for compound **1o**.
