# Supplementary material for: One-pot synthesis of tetracyclic fused imidazo[1,2-a]pyridines via a three-component reaction
Source: Beilstein J Org Chem. 2016 Jul 18;12:1487–92. doi: 10.3762/bjoc.12.145 (PMC4979633; doi:10.3762/bjoc.12.145)
Supplement: File 1 — Experimental procedures, characterization and spectral data for synthesized compounds and X-ray data for compound 1o. [file Beilstein_J_Org_Chem-12-1487-s001.pdf]

# Supporting Information

# for

# One-pot synthesis of tetracyclic fused imidazo[1,2-a]pyridines via a three-component reaction

Bo Yang, Chuanye Tao, Taofeng Shao, Jianxian Gong* and Chao Che*

Address: Laboratory of Chemical Genomics, Engineering Laboratory for Chiral Drug Synthesis, School of Chemical Biology and Biotechnology, Peking University Shenzhen Graduate School, Shenzhen 518055, China

Email: Jianxian Gong* - gongjx@pku.edu.cn; Chao Che* chec@pkusz.edu.cn

*Corresponding author

Experimental procedures, characterization and spectral data for synthesized compounds and X-ray data for compound **1o**

## General Information

Unless noted otherwise, all reactions were performed under a nitrogen atmosphere, and materials obtained from commercial suppliers were used without further purification. $^{1}$H NMR spectra were recorded on a 300 MHz, 400 MHz or 500 MHz spectrometer using residual solvent signal (DMSO δ = 2.50 ppm) as internal standard. All coupling constants are reported in hertz (Hz). $^{13}$C NMR spectra were recorded on the same instruments, and chemical shifts were measured relative to solvent resonances (DMSO δ = 39.9 ppm). High-resolution mass spectra were obtained on a quadrupole time-of-flight (QqTOF) mass spectrometer utilizing electrospray ionization (ESI) method.

## Typical procedure for the multicomponent reaction

To a solution of isatin **3** (1.0 mmol), 2-aminopyridine **2** (1.35 mmol) and isocyanide **4** (1.35 mmol) in 4 mL of *n*-butyl alcohol was added $HClO_4$ (1.0 mmol), and the reaction mixture was stirred under reflux for 8 h. After cooling to room temperature, the precipitate was collected by filtration, rinsed with ethanol and dried to afford the target compound **1**.

*13-(tert-Butylamino)-6-oxo-5,6-dihydropyrido[2',1':2,3]imidazo[1,5-c]quinazolin-12-ium perchlorate* ***(1a):*** 171 mg (42%) as a slight yellow solid; mp 252.3−253.7°C; $^1$H NMR (400 MHz, DMSO-$d_6$) $\delta$ 1.25 (*s*, 9H), 5.63 (*s*, 1H), 7.35 (*d*, *J* = 8.4 Hz, 1H), 7.41 (*t*, *J* = 7.2 Hz, 1H), 7.61-7.65 (*m*, 1H), 7.88-7.92 (*m*, 1H), 8.27-8.32 (*m*, 1H), 8.70 (*d*, *J* = 7.2 Hz, 1H), 8.95 (*d*, *J* = 8.8 Hz, 1H), 9.14 (*d*, *J* = 6.8 Hz, 1H), 12.29 (*s*, 1H); $^{13}$C NMR (75 MHz, DMSO-$d_6$) $\delta$ 30.4, 58.1, 110.7, 115.2, 117.5, 119.8, 123.1, 123.3, 125.3, 125.6, 127.3, 131.9, 134.7, 135.8, 137.2, 145.0; HRMS (*m/z*, ESI) calc. for $C_{18}H_{19}N_4O$ (+) 307.1553, found 307.1552.

*13-(tert-Butylamino)-10-chloro-6-oxo-5,6-dihydropyrido[2',1':2,3]imidazo[1,5-c]quinazolin-12-ium perchlorate (**1b**):* 234 mg (53%) as a slight yellow solid; mp 248.8−250.6 °C; $^1$H NMR (400 MHz, DMSO-$d_6$) $\delta$ 1.25 (*s*, 9H), 5.69 (*s*, 1H), 7.40-7.43 (*m*, 2H), 7.62-7.66 (*m*, 1H), 8.35 (*dd*, *J* = 2.0, 9.6 Hz, 1H), 8.69 (*dd*, *J* = 1.2, 8.4 Hz, 1H), 8.95 (*d*, *J* = 9.6 Hz, 1H), 9.40 (*d*, *J* = 1.2 Hz, 1H), 12.53 (*s*, 1H); $^{13}$C NMR (100 MHz, DMSO-$d_6$) $\delta$ 30.2, 58.8, 110.5, 116.1, 116.8, 123.9, 124.2, 125.7, 125.8, 126.8, 132.4, 133.8, 135.9, 136.3, 144.4; HRMS (*m/z*, ESI) calc. for $C_{18}H_{18}N_4OCl$ (+) 341.1164, found 341.1152.

*13-(tert-Butylamino)-10-methyl-6-oxo-5,6-dihydropyrido[2',1':2,3]imidazo[1,5-c]quinazolin-12-ium perchlorate (**1c**):* 160 mg (38%) as a brown solid; mp 258.9−260.4 °C; $^1$H NMR (400 MHz, DMSO-$d_6$) $\delta$ 1.25 (*s*, 9H), 2.59 (*s*, 3H), 5.54 (*s*, 1H), 7.34-7.41 (*m*, 2H), 7.59-7.63 (*m*, 1H), 8.15 (*d*, *J* = 9.2 Hz, 1H), 8.68 (*d*, *J* = 8.0 Hz, 1H), 8.84 (*d*, *J* = 9.2 Hz, 1H), 8.95 (*s*, 1H), 12.26 (*s*, 1H); $^{13}$C NMR (100 MHz, DMSO-$d_6$) $\delta$ 18.2, 30.4, 58.3, 110.7, 114.5, 116.4, 123.5, 123.8, 125.0, 125.1, 125.8, 130.5, 132.2, 133.8, 135.5, 138.7, 144.7; HRMS (*m/z*, ESI) calc. for $C_{19}H_{21}N_4O$ (+) 321.1710, found 321.1700.

*10-Bromo-13-(tert-butylamino)-6-oxo-5,6-dihydropyrido[2',1':2,3]imidazo[1,5-c]quinazolin-12-ium perchlorate (**1d**):* 267 mg (55%) as a slight yellow solid; mp 267.8−269.1 °C; $^1$H NMR (300 MHz, DMSO-$d_6$) $\delta$ 1.24 (*s*, 9H), 5.56 (*s*, 1H), 7.36-7.45 (*m*, 2H), 7.62-7.67 (*m*, 1H), 8.43 (*dd*, *J* = 1.8, 9.6 Hz, 1H), 8.69 (*d*, *J* = 7.2 Hz, 1H), 8.87 (*d*, *J* = 9.6 Hz, 1H), 9.38 (*s*,

1H), 12.45 (*s*, 1H); $^{13}$C NMR (100 MHz, DMSO-$d_6$) $\delta$ 30.2, 58.7, 110.4, 114.2, 116.2, 116.6, 123.9, 124.1, 125.5, 125.9, 127.3, 132.6, 134.1, 135.4, 138.8, 144.4; HRMS (*m/z,* ESI) calc. for $C_{18}H_{18}N_4OBr$ (+) 385.0659, found 385.0653.

13-(*tert*-Butylamino)-2-methoxy-10-methyl-6-oxo-5,6-dihydropyrido[2',1':2,3]imidazo[1,5-*c*]quin-azolin-12-ium perchlorate (**1e**): 225 mg (50%) as a grey solid; mp 270.8–272.7 °C; $^1$H NMR (300 MHz, DMSO-$d_6$) $\delta$ 1.27 (*s*, 9H), 2.59 (*s*, 3H), 3.87 (*s*, 3H), 5.64 (*s*, 1H), 7.26-7.28 (*m*, 2H), 8.13-8.17 (*m*, 2H), 8.84 (*d*, *J* = 9.3 Hz, 1H), 8.95 (*s*, 1H), 12.17 (*s*, 1H); $^{13}$C NMR (100 MHz, DMSO-$d_6$) $\delta$ 18.2, 30.5, 56.2, 58.1, 108.8, 111.4, 114.5, 117.8, 120.0, 123.5, 124.9, 125.1, 129.2, 130.5, 133.9, 138.6, 144.4, 155.4; HRMS (*m/z,* ESI) calc. for $C_{20}H_{23}N_4O_2$ (+) 351.1816, found 351.1801.

*13-(tert-Butylamino)-10-chloro-2-methoxy-6-oxo-5,6-dihydropyrido[2',1':2,3]imidazo[1,5-c]quinazolin-12-ium perchlorate (**1f**):* 184 mg (39%) as a yellow solid; mp 238.4–240.5 °C; $^1$H NMR (400 MHz, DMSO-$d_6$) $\delta$ 1.27 (*s*, 9H), 3.88 (*s*, 3H), 5.66 (*s*, 1H), 7.28-7.34 (*m*, 2H), 8.18 (*d*, *J* = 2.8 Hz, 1H), 8.34 (*dd*, *J* = 2.0, 9.6 Hz, 1H), 8.96 (*d*, *J* = 9.6 Hz, 1H), 9.33 (*d*, *J* = 1.6 Hz, 1H) 12.20 (*br*, 1H); $^{13}$C NMR (100 MHz, DMSO-$d_6$) $\delta$ 30.3, 56.2, 58.5, 108.6, 111.0, 116.2, 118.1, 120.5, 124.1, 125.2, 125.7, 127.0, 129.5, 133.9, 136.4, 144.2, 155.5; HRMS (*m/z,* ESI) calc. for $C_{19}H_{20}N_4O_2Cl$ (+) 371.1269, found 371.1277.

*10-Bromo-13-(tert-butylamino)-2-methoxy-6-oxo-5,6-dihydropyrido[2',1':2,3]imidazo[1,5-c]quinazolin -12-ium perchlorate (**1g**):* 247 mg (48%) as a brown solid; mp 249.5–251.3 °C; $^1$H NMR (300 MHz, DMSO-$d_6$) $\delta$ 1.26 (*s*, 9H), 3.88 (*s*, 3H), 5.65 (*s*, 1H), 7.28-7.32 (*m*, 2H), 8.17 (*d*, *J* = 2.1 Hz, 1H), 8.42 (*dd*, *J* = 1.5, 9.6 Hz, 1H), 8.87 (*d*, *J* = 9.6 Hz, 1H), 9.37 (*s*, 1H) 12.35 (*s*, 1H); $^{13}$C NMR (125 MHz, DMSO-$d_6$) $\delta$ 30.4, 56.3, 58.5, 108.7, 111.1, 114.2, 116.3, 118.1, 120.5, 123.9, 125.5, 127.2, 129.4, 134.2, 138.8, 144.2, 155.6; HRMS (*m/z,* ESI) calc. for $C_{19}H_{20}N_4O_2Br$ (+) 415.0764, found 415.0764.

*13-(tert-Butylamino)-2-chloro-6-oxo-5,6-dihydropyrido[2',1':2,3]imidazo[1,5-c]quinazolin-12-ium perchlorate (**1h**):* 163 mg (37%) as a slight yellow solid; mp 285.7–287.4 °C; $^1$H NMR (300 MHz, DMSO-$d_6$) $\delta$ 1.25 (*s*, 9H), 5.77 (*s*, 1H), 7.35 (*d*, *J* = 9.0 Hz, 1H), 7.67 (*dd*, *J* = 2.4, 8.7 Hz, 1H), 7.89 (*t*, *J* = 6.9 Hz, 1H), 8.32 (*t*, *J* = 7.8 Hz, 1H), 8.71 (*d*, *J* = 2.1 Hz, 1H), 8.95 (*d*, *J* = 9.0 Hz, 1H), 9.17 (*d*, *J* = 6.6 Hz, 1H); $^{13}$C NMR (75 MHz, DMSO-$d_6$) $\delta$ 30.1, 58.1, 111.9, 114.8, 118.3, 120.0, 123.6, 124.0, 124.5, 127.2, 131.7, 132.3, 134.4, 134.8, 136.5, 144.4; HRMS (*m/z*, ESI) calc. for $C_{18}H_{18}N_4OCl$ (+) 341.1164, found 341.1156.

*10-Bromo-13-(tert-butylamino)-2-chloro-6-oxo-5,6-dihydropyrido[2',1':2,3]imidazo[1,5-c]quinazolin-12-ium perchlorate (**1i**):* 213 mg (41%) as a grey solid; mp 265.6–267.3 °C; $^1$H NMR (400 MHz, DMSO-$d_6$) $\delta$ 1.25 (*s*, 9H), 5.68 (*s*, 1H), 7.38 (*d*, *J* = 8.4 Hz, 1H), 7.71 (*d*, *J* = 8.4 Hz, 1H), 8.47 (*d*, *J* = 9.6 Hz, 1H), 8.70 (*s*, 1H), 8.87 (*d*, *J* = 9.2 Hz, 1H), 9.40 (*s*, 1H), 12.57 (*s*, 1H); $^{13}$C NMR (100 MHz, DMSO-$d_6$) $\delta$ 30.2, 58.9, 111.9, 114.6, 116.2, 118.5, 124.4, 124.5, 124.8, 127.5, 127.9, 132.4, 134.2, 134.5, 139.5, 144.2; HRMS (*m/z*, ESI) calc. for $C_{18}H_{17}N_4OClBr$ (+) 419.0269, found 419.0251.

*2-Bromo-13-(tert-butylamino)-6-oxo-5,6-dihydropyrido[2',1':2,3]imidazo[1,5-c]quinazolin-12-ium perchlorate (**1j**):* 189 mg (39%) as a slight yellow solid; mp 284.8–286.5 °C; $^1$H NMR (400 MHz, DMSO-$d_6$) $\delta$ 1.25 (*s*, 9H), 5.76 (*s*, 1H), 7.31 (*s*, 1H), 7.81-7.92 (*m*, 2H), 8.33 (*s*, 1H), 8.86-8.96 (*m*, 2H), 9.18 (*s*, 1H), 12.44 (*s*, 1H); $^{13}$C NMR (100 MHz, DMSO-$d_6$) $\delta$ 30.4, 58.4, 115.2, 115.5, 118.5, 120.4, 121.6, 123.8, 124.4, 127.7, 134.7, 135.2, 137.2, 137.7, 144.6; HRMS (*m/z*, ESI) calc. for $C_{18}H_{18}N_4OBr$ (+) 385.0659, found 385.0658.

*2,10-Dibromo-13-(tert-butylamino)-6-oxo-5,6-dihydropyrido[2',1':2,3]imidazo[1,5-c]quinazolin-12-ium perchlorate (**1k**):* 197 mg (35%) as a slight yellow solid; mp 259.7–261.6 °C; $^1$H NMR (400 MHz, DMSO-$d_6$) $\delta$ 1.25 (*s*, 9H), 5.68 (*s*, 1H), 7.31 (*d*, *J* = 8.8 Hz, 1H), 7.82 (*d*, *J* = 7.2 Hz, 1H), 8.47 (*d*, *J* = 9.2 Hz, 1H), 8.84-8.89 (*m*, 2H), 9.39 (*s*, 1H), 12.56 (*s*, 1H); $^{13}$C NMR (100 MHz, DMSO-$d_6$) $\delta$ 30.2, 58.9, 112.3, 114.5, 115.6, 116.2, 118.7, 124.2, 124.5, 127.4, 127.8, 134.2, 134.7, 135.1, 139.5, 144.2; HRMS (*m/z*, ESI) calc. for $C_{18}H_{17}N_4OBr_2$ (+)

462.9764, found 462.9748.

*13-(tert-Butylamino)-10-chloro-2,4-dimethyl-6-oxo-5,6-dihydropyrido[2',1':2,3]imidazo[1,5-c]quinazolin-12-ium perchlorate* (***1l***)*:* 188 mg (40%) as a brown solid; mp 242.3–244.2 °C; $^1$H NMR (300 MHz, DMSO-$d_6$) $\delta$ 1.24 (*s*, 9H), 2.38 (*s*, 3H), 2.43 (*s*, 3H), 5.54 (*s*, 1H), 7.34 (*s*, 1H), 8.33 (*d*, *J* = 9.9 Hz, 1H), 8.43 (*s*, 1H), 8.95 (*d*, *J* = 9.3 Hz, 1H), 9.32 (*s*, 1H) 11.50 (*s*, 1H); $^{13}$C NMR (100 MHz, DMSO-$d_6$) $\delta$ 18.0, 20.8, 30.3, 58.7, 110.5, 116.3, 123.4, 123.8, 125.2, 125.4, 125.8, 127.1, 131.8, 132.9, 133.7, 134.9, 136.3, 144.7; HRMS (*m/z*, ESI) calc. for $C_{20}H_{22}N_4OCl$ (+) 369.1477, found 369.1477.

*13-(tert-Butylamino)-10-chloro-4-fluoro-6-oxo-5,6-dihydropyrido[2',1':2,3]imiazo[1,5-c]quinazolin-12-ium perchlorate* (***1m***)*:* 174 mg (38%) as a yellow solid; mp 236.5–238.4 °C; $^1$H NMR (300 MHz, DMSO-$d_6$) $\delta$ 1.24 (*s*, 9H), 5.60 (*s*, 1H), 7.43-7.45 (*m*, 1H), 7.56-7.62 (*m*, 1H), 8.39 (*dd*, *J* = 1.8, 9.3 Hz, 1H), 8.50 (*d*, *J* = 7.8 Hz, 1H), 8.96 (*d*, *J* = 9.6 Hz, 1H), 9.36 (*d*, *J* = 1.5 Hz, 1H), 12.57 (*s*, 1H); $^{13}$C NMR (100 MHz, DMSO-$d_6$) $\delta$ 30.2, 58.8, 112.6, 116.2, 118.1, 118.2, 121.6, 124.3, 124.5, 124.6, 125.1, 125.5, 127.3, 134.1, 137.1, 144.3; HRMS (*m/z*, ESI) calc. for $C_{18}H_{17}N_4OClF$ (+) 359.1069, found 359.1069.

*13-(Cyclohexylamino)-10-methyl-6-oxo-5,6-dihydropyrido[2',1':2,3]imidazo[1,5-c]quinazolin-12-ium perchlorate* (***1n***)*:* 232 mg (52%) as a grey solid; mp 244.9–246.6 °C; $^1$H NMR (400 MHz, DMSO-$d_6$) $\delta$ 1.13-1.22 (*m*, 3H), 1.38-1.43 (*m*, 2H), 1.56-1.59 (*m*, 1H), 1.65-1.72 (*m*, 2H), 1.92-1.94 (*m*, 2H), 2.60 (*s*, 3H), 3.04-3.07 (*m*, 1H), 5.70 (*d*, *J* = 6.0 Hz, 1H), 7.35 (*d*, *J* = 8.0 Hz, 1H), 7.44 (*t*, *J* = 7.6 Hz, 1H), 7.62 (*t*, *J* = 8.0 Hz, 1H), 8.14 (*d*, *J* = 9.2 Hz, 1H), 8.44 (*d*, *J* = 8.0 Hz, 1H), 8.82 (*d*, *J* = 9.2 Hz, 1H), 8.88 (*s*, 1H), 12.25 (*s*, 1H); $^{13}$C NMR (100 MHz, DMSO-$d_6$) $\delta$ 18.3, 25.1, 25.6, 33.6, 57.1, 110.8, 114.5, 116.5, 122.7, 124.5, 124.7, 124.9, 131.0, 131.9, 133.3, 135.2, 138.5, 144.6; HRMS (*m/z*, ESI) calc. for $C_{21}H_{23}N_4O$ (+) 347.1866, found 347.1852.

*10-Chloro-13-(cyclohexylamino)-6-oxo-5,6-dihydropyrido[2',1':2,3]imidazo[1,5-c]quin-azolin-12-ium perchlorate (**1o**):* 224 mg (48%) as a brown solid; mp 258.7–260.4 °C; $^1$H NMR (400 MHz, DMSO-$d_6$) $\delta$ 1.13-1.17 (*m*, 3H), 1.36-1.41 (*m*, 2H), 1.56-1.59 (*m*, 1H), 1.65-1.72 (*m*, 2H), 1.92-1.96 (*m*, 2H), 3.06 (*m*, 1H), 5.74 (*d*, *J* = 4.4 Hz, 1H), 7.38 (*d*, *J* = 8.0 Hz, 1H), 7.48 (*t*, *J* = 7.6 Hz, 1H), 7.64 (*d*, *J* = 7.6 Hz, 1H), 8.35 (*dd*, *J* = 1.6, 9.6 Hz, 1H), 8.43 (*d*, *J* = 8.0 Hz, 1H), 8.92 (*d*, *J* = 9.6 Hz, 1H), 9.32 (*s*, 1H), 12.44 (*s*, 1H); $^{13}$C NMR (125 MHz, DMSO-$d_6$) $\delta$ 25.2, 25.7, 33.6, 57.4, 110.5, 116.1, 116.7, 123.4, 124.8, 124.9, 125.1, 125.5, 127.5, 132.4, 133.3, 135.3, 136.4, 144.4; HRMS (*m/z*, ESI) calc. for $C_{20}H_{20}N_4OCl$ (+) 367.1320, found 367.1313.

*10-Bromo-13-(cyclohexylamino)-6-oxo-5,6-dihydropyrido[2',1':2,3]imidazo[1,5-c]quin-azolin-12-ium perchlorate (**1p**):* 230 mg (45%) as a brown solid; mp 286.6–288.7 °C; $^1$H NMR (300 MHz, DMSO-$d_6$) $\delta$ 1.12-1.21 (*m*, 3H), 1.38-1.44 (*m*, 2H), 1.56-1.57 (*m*, 1H), 1.65-1.73 (*m*, 2H), 1.92-1.95 (*m*, 2H), 3.03-3.04 (*m*, 1H), 5.73 (*d*, *J* = 6.9 Hz, 1H), 7.38 (*d*, *J* = 8.1 Hz, 1H), 7.45-7.50 (*m*, 1H), 7.63-7.68 (*m*, 1H), 8.41-8.45 (*m*, 2H), 8.84 (*d*, *J* = 9.6 Hz, 1H), 9.36 (*s*, 1H), 12.43 (*s*, 1H); $^{13}$C NMR (125 MHz, DMSO-$d_6$) $\delta$ 25.2, 25.7, 33.6, 57.5, 110.5, 114.7, 116.2, 116.7, 123.1, 124.8, 125.1, 125.4, 126.7, 132.4, 133.6, 135.3, 138.7, 144.4; HRMS (*m/z*, ESI) calc. for $C_{20}H_{20}N_4OBr$ (+) 411.0815, found 411.0800.

*10-Chloro-13-(cyclohexylamino)-2-methoxy-6-oxo-5,6-dihydropyrido[2',1':2,3]imi-dazo[1,5-c]quinazolin-12-ium perchlorate (**1q**):* 234 mg (47%) as a brown solid; mp 269.5–271.2 °C; $^1$H NMR (300 MHz, DMSO-$d_6$) $\delta$ 1.13-1.22 (*m*, 3H), 1.38-1.43 (*m*, 2H), 1.56-1.59 (*m*, 1H), 1.65-1.72 (*m*, 2H), 1.92-1.95 (*m*, 2H), 3.07-3.10 (*m*, 1H), 3.88 (*s*, 3H), 5.81 (*d*, *J* = 6.6 Hz, 1H), 7.28-7.36 (*m*, 2H), 7.90 (*d*, *J* = 2.1 Hz, 1H), 8.33 (*dd*, *J* = 1.5, 9.3 Hz, 1H), 8.92 (*d*, *J* = 9.6Hz, 1H), 9.33 (*s*, 1H), 12.34 (*br*, 1H); $^{13}$C NMR (75 MHz, DMSO-$d_6$) $\delta$ 25.1, 25.6, 33.7, 56.1, 57.5, 107.6, 111.0, 116.1, 118.1, 120.3, 123.0, 124.7, 125.5, 127.3, 129.0, 133.2, 136.1, 144.1, 156.0; HRMS (*m/z*, ESI) calc. for $C_{21}H_{22}N_4O_2Cl$ (+) 397.1426, found 397.1419.

*10-Bromo-13-(cyclohexylamino)-2-methoxy-6-oxo-5,6-dihydropyrido[2',1':2,3]imidazo[1,5-c]quinazolin-12-ium perchlorate (**1r**):* 206 mg (38%) as a green solid; mp 275.2–277.3 °C; $^{1}$H NMR (500 MHz, DMSO-$d_6$) $\delta$ 1.15-1.22 (*m*, 3H), 1.40-1.44 (*m*, 2H), 1.57-1.60 (*m*, 1H), 1.69-1.72 (*m*, 2H), 1.92-1.96 (*m*, 2H), 3.08-3.10 (*m*, 1H), 3.88 (*s*, 3H), 5.78 (*d*, *J* = 6.2 Hz, 1H), 7.28-7.35 (*m*, 2H), 7.90 (*s*, 1H), 8.41 (*d*, *J* = 9.5 Hz, 1H), 8.85 (*d*, *J* = 9.5 Hz, 1H), 9.36 (*s*, 1H), 12.31 (*s*, 1H); $^{13}$C NMR (125 MHz, DMSO-$d_6$) $\delta$ 25.1, 25.7, 33.8, 56.2, 57.7, 107.8, 111.1, 114.6, 116.2, 118.2, 120.3, 123.0, 125.4, 126.6, 129.1, 133.6, 138.5, 144.1, 156.2; HRMS (*m/z*, ESI) calc. for $C_{21}H_{22}N_4O_2Br$ (+) 441.0921, found 441.0900.

*2,10-Dichloro-13-(cyclohexylamino)-6-oxo-5,6-dihydropyrido[2',1':2,3]imidazo[1,5-c]quinazolin-12-ium perchlorate (**1s**):* 256 mg (51%) as a slight purple solid; mp 249.1–250.8 °C; $^{1}$H NMR (500 MHz, DMSO-$d_6$) $\delta$ 1.16-1.22 (*m*, 3H), 1.38-1.42 (*m*, 2H), 1.56-1.59 (*m*, 1H), 1.65-1.72 (*m*, 2H), 1.92-1.94 (*m*, 2H), 3.03-3.06 (*m*, 1H), 5.88 (*d*, *J* = 6.8 Hz, 1H), 7.39 (*d*, *J* = 8.8 Hz, 1H), 7.71-7.73 (*m*, 1H), 8.38-8.44 (*m*, 2H), 8.92 (*d*, *J* = 9.6 Hz, 1H), 9.31 (*s*, 1H), 12.55 (*s*, 1H); $^{13}$C NMR (125 MHz, DMSO-$d_6$) $\delta$ 25.1, 25.7, 33.8, 57.6, 112.0, 116.1, 118.7, 122.2, 124.2, 125.1, 126.1, 127.7, 128.6, 132.2, 133.5, 134.3, 137.0, 144.2; HRMS (*m/z*, ESI) calc. for $C_{20}H_{19}N_4OCl_2$ (+) 401.0930, found 401.0917.

*10-Bromo-2-chloro-13-(cyclohexylamino)-6-oxo-5,6-dihydropyrido[2',1':2,3]imidazo[1,5-c]quinazolin-12-ium perchlorate (**1t**):* 218 mg (40%) as a slight purple solid; mp 278.8–280.8 °C; $^{1}$H NMR (400 MHz, DMSO-$d_6$) $\delta$ 1.12-1.22 (*m*, 3H), 1.35-1.43 (*m*, 2H), 1.56-1.59 (*m*, 1H), 1.65-1.72 (*m*, 2H), 1.92-1.99 (*m*, 2H), 3.02-3.06 (*m*, 1H), 5.88 (*d*, *J* = 6.8 Hz, 1H), 7.38 (*d*, *J* = 8.8 Hz, 1H), 7.71 (*dd*, *J* = 2.4, 8.8 Hz, 1H), 8.42-8.48 (*m*, 2H), 8.84 (*d*, *J* = 9.6 Hz, 1H), 9.36 (*d*, *J* = 1.2 Hz, 1H), 12.56 (*s*, 1H); $^{13}$C NMR (100 MHz, DMSO-$d_6$) $\delta$ 25.1, 25.6, 33.7, 57.6, 112.0, 114.8, 116.1, 118.6, 121.7, 124.0, 125.9, 126.9, 128.5, 132.0, 133.6, 134.2, 139.2, 144.2; HRMS (*m/z*, ESI) calc. for $C_{20}H_{19}N_4OClBr$ (+) 445.0425, found 445.0419.

*2-Bromo-10-chloro-13-(cyclohexylamino)-6-oxo-5,6-dihydropyrido[2',1':2,3]imidazo[1,5-c]quinazolin-12-ium perchlorate (**1u**):* 186 mg (34%) as a slight yellow solid; mp 286.8–288.7 °C; $^{1}H$ NMR (300 MHz, DMSO-$d_6$) $\delta$ 1.12-1.21 (*m*, 3H), 1.38-1.42 (*m*, 2H), 1.57-1.58 (*m*, 1H), 1.69-1.73 (*m*, 2H), 1.91-1.95 (*m*, 2H), 3.04 (*m*, 1H), 5.90 (*d*, *J* = 6.9 Hz, 1H), 7.31 (*d*, *J* = 8.7 Hz, 1H), 7.82 (*dd*, *J* = 2.1, 8.7 Hz, 1H), 8.37 (*dd*, *J* = 1.8, 9.6 Hz, 1H), 8.56 (*d*, *J* = 1.8 Hz, 1H), 8.91 (*d*, *J* = 9.6 Hz, 1H), 9.32 (*d*, *J* = 1.2 Hz, 1H), 12.57 (*s*, 1H); $^{13}C$ NMR (75 MHz, DMSO-$d_6$) $\delta$ 25.1, 25.6, 33.7, 57.5, 112.4, 116.0, 116.2, 118.7, 121.8, 125.0, 126.1, 127.0, 127.6, 133.3, 134.5, 134.8, 136.8, 144.2; HRMS (*m/z*, ESI) calc. for $C_{20}H_{19}N_4OClBr$ (+) 445.0425, found 445.0430.

*2,10-Dibromo-13-(cyclohexylamino)-6-oxo-5,6-dihydropyrido[2',1':2,3]imidazo[1,5-c]quinazolin-12-ium perchlorate (**1v**):* 219 mg (37%) as a green solid; mp 278.2–280.3 °C; $^{1}H$ NMR (500 MHz, DMSO-$d_6$) $\delta$ 1.14-1.21 (*m*, 3H), 1.38-1.41(*m*, 2H), 1.57-1.58 (*m*, 1H), 1.69-1.73 (*m*, 2H), 1.91-1.94 (*m*, 2H), 3.03-3.05 (*m*, 1H), 5.86 (*d*, *J* = 7.0 Hz, 1H), 7.32 (*d*, *J* = 8.8 Hz, 1H), 7.82 (*d*, *J* = 8.9 Hz, 1H), 8.45 (*d*, *J* = 9.5 Hz, 1H), 8.55 (*s*, 1H), 8.84 (*d*, *J* = 9.5 Hz, 1H), 9.35 (*s*, 1H), 12.53 (*s*, 1H); $^{13}C$ NMR (100 MHz, DMSO-$d_6$) $\delta$ 25.1, 25.6, 33.7, 57.6, 112.4, 114.8, 116.1, 116.2, 118.8, 121.6, 125.9, 126.8, 127.0, 133.5, 134.7, 139.1, 144.2; HRMS (*m/z*, ESI) calc. for $C_{20}H_{19}N_4OBr_2$ (+) 488.9920, found 488.9906.

*13-(Cyclohexylamino)-2-iodo-10-methyl-6-oxo-5,6-dihydropyrido[2',1':2,3]imidazo[1,5-c]quinazolin-12-ium perchlorate (**1w**):* 206 mg (36%) as a slight yellow solid; mp 285.7–287.8 °C; $^{1}H$ NMR (400 MHz, DMSO-$d_6$) $\delta$ 1.15-1.22 (*m*, 3H), 1.40-1.49 (*m*, 2H), 1.58-1.60 (*m*, 1H), 1.71-1.78 (*m*, 2H), 1.92-1.95 (*m*, 2H), 2.60 (*s*, 3H), 3.01-3.04 (*m*, 1H), 5.83 (*d*, *J* = 6.4 Hz, 1H), 7.14 (*d*, *J* = 8.4 Hz, 1H), 7.91 (*d*, *J* = 8.8 Hz, 1H), 8.16 (*d*, *J* = 9.2 Hz, 1H), 8.75 (*s*, 1H), 8.81 (*d*, *J* = 9.2 Hz, 1H), 8.89 (*s*, 1H), 12.33 (*s*, 1H); $^{13}C$ NMR (100 MHz, DMSO-$d_6$) $\delta$ 18.3, 25.1, 25.7, 33.8, 57.3, 87.7, 113.0, 114.5, 118.5, 121.1, 124.6, 125.3, 131.2, 132.9, 133.4, 134.8, 138.9, 139.9, 144.4; HRMS (*m/z*, ESI) calc. for $C_{21}H_{22}N_4OI$ (+) 473.0833, found 473.0811.

*10-Bromo-13-(cyclohexylamino)-4-fluoro-6-oxo-5,6-dihydropyrido[2',1':2,3]imidazo[1,5-c]quinazolin-12-ium perchlorate (**1x**):* 175 mg (33%) as a slight yellow solid; mp 254.6–256.7 °C; $^1$H NMR (400 MHz, DMSO-$d_6$) $\delta$ 1.14-1.22 (*m*, 3H), 1.38-1.444 (*m*, 2H), 1.58-1.60 (*m*, 1H), 1.67-1.76 (*m*, 2H), 1.91-1.94 (*m*, 2H), 3.03-3.05 (*m*, 1H), 5.77 (*d*, *J* = 6.4 Hz, 1H), 7.46-7.51 (*m*, 1H), 7.57-7.62 (*m*, 1H), 8.23 (*d*, *J* = 7.6 Hz, 1H), 8.46 (*d*, *J* = 9.2 Hz, 1H), 8.87 (*d*, *J* = 9.2 Hz, 1H), 9.39 (*s*, 1H), 12.53 (*s*, 1H); $^{13}$C NMR (100 MHz, DMSO-$d_6$) $\delta$ 25.1, 25.6, 33.5, 57.5, 112.7, 114.8, 116.2, 117.8, 118.0, 120.7, 123.8, 124.0, 125.2, 125.9, 126.9, 133.7, 139.2, 144.2; HRMS (*m/z*, ESI) calc. for $C_{20}H_{19}N_4OBrF$ (+) 429.0721, found 429.0725.

*10-Chloro-13-(cyclohexylamino)-2,4-dimethyl-6-oxo-5,6-dihydropyrido[2',1':2,3]imidazo[1,5-c]quinazolin-12-ium perchlorate (**1y**):* 188 mg (38%) as a yellow solid; mp 212.4–214.2 °C; $^1$H NMR (300 MHz, DMSO-$d_6$) $\delta$ 1.14-1.22 (*m*, 3H), 1.38-1.43 (*m*, 2H), 1.54-1.60 (*m*, 1H), 1.66-1.72 (*m*, 2H), 1.89-1.93 (*m*, 2H), 2.38 (*s*, 3H), 2.42 (*s*, 3H), 3.01-3.04 (*m*, 1H), 5.66 (*d*, *J* = 6.9 Hz, 1H), 7.31 (*s*, 1H), 8.10 (*s*, 1H), 8.24 (*dd*, *J* = 1.8, 9.3 Hz, 1H), 8.95 (*d*, *J* = 9.3 Hz, 1H), 9.26 (*s*, 1H); $^{13}$C NMR (100 MHz, DMSO-$d_6$) $\delta$ 18.1, 21.0, 25.1, 25.6, 33.7, 57.4, 110.4, 116.2, 122.4, 123.8, 124.2, 124.8, 126.4, 127.1, 132.6, 132.7, 134.3, 135.0, 144.9; HRMS (*m/z*, ESI) calc. for $C_{22}H_{24}N_4OCl$ (+) 395.1633, found 395.1632.

**Note:** Yields and NMR shifts of the compounds (**1a**, **1e**, **1f**, **1l**, **1m**, **1r**, **1u**, **1v** and **1w**) were already published in CN105017253 A.

# $^{1}H$ NMR and $^{13}C$ NMR spectra

**1a**

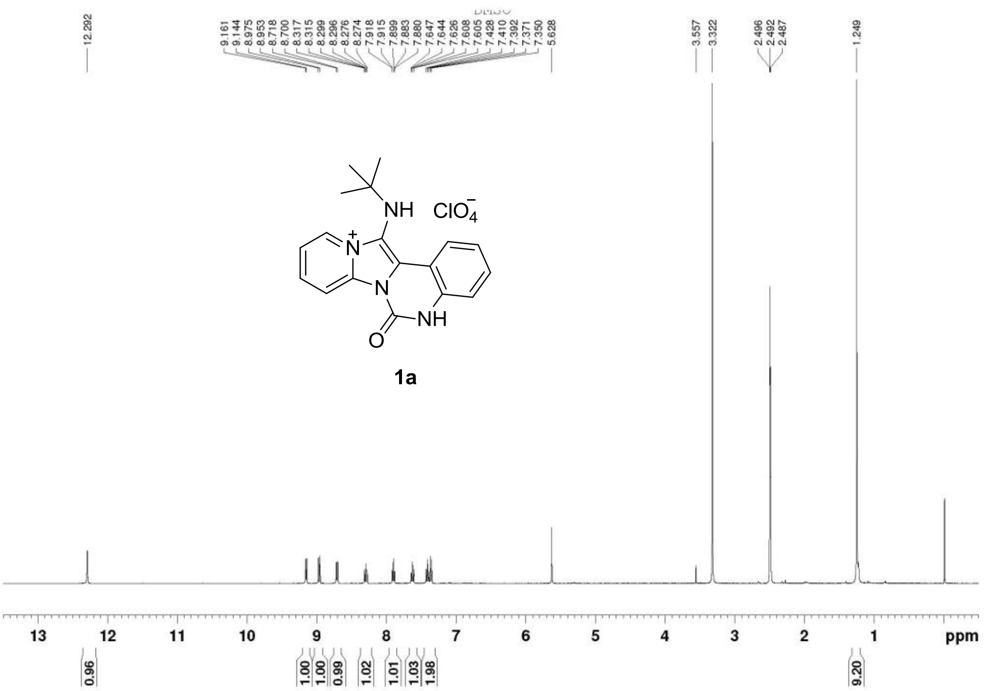

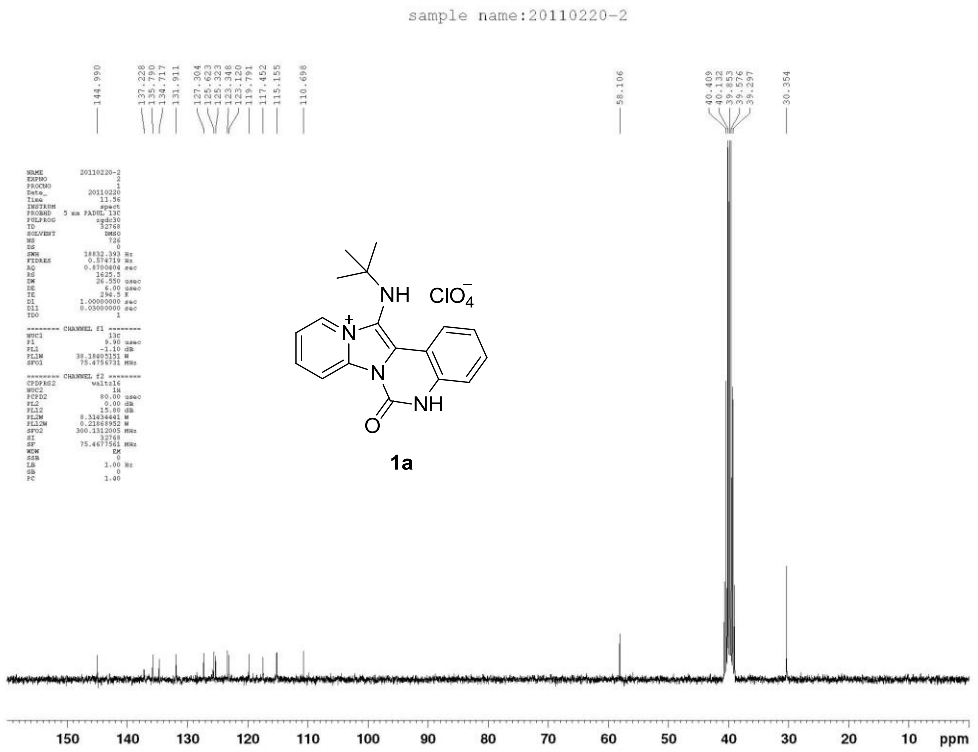

sample name:20110220-2
144.990
137.228
135.790
134.717
131.911
127.304
125.623
125.323
123.348
123.120
119.791
117.452
115.155
110.698
58.106
40.409
40.132
39.853
39.576
39.297
30.354
NH
ClO4−
N+
N
NH
O
1a
150 140 130 120 110 100 90 80 70 60 50 40 30 20 10 ppm


**1b**

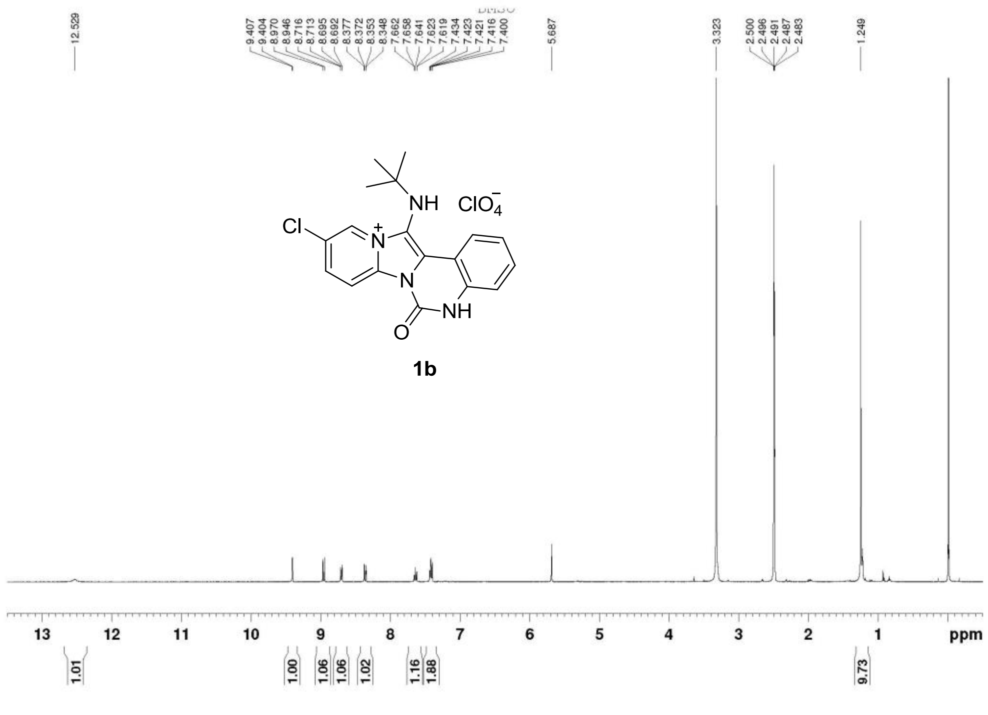

12.529
9.407
9.404
8.970
8.946
8.716
8.713
8.695
8.692
8.377
8.372
8.353
8.348
7.662
7.658
7.641
7.623
7.619
7.434
7.423
7.421
7.416
7.400
5.687
3.323
2.500
2.496
2.491
2.487
2.483
1.249
NH
ClO4−
Cl
N+
N
NH
O
1b
13 12 11 10 9 8 7 6 5 4 3 2 1 ppm
1.01
1.00
1.06
1.06
1.02
1.16
1.88
9.73

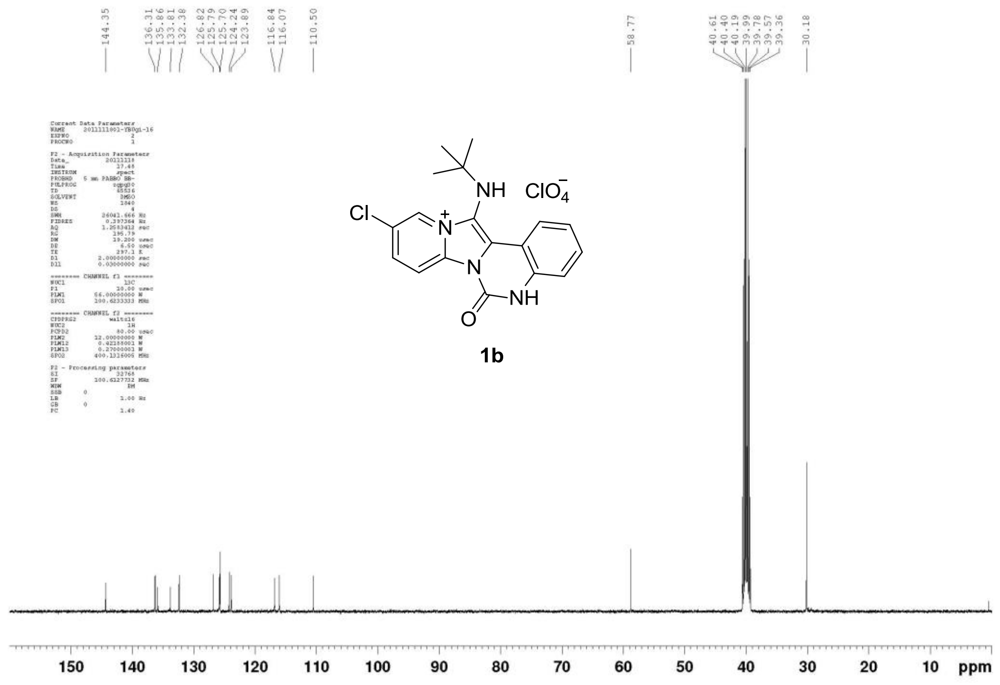


**1c**

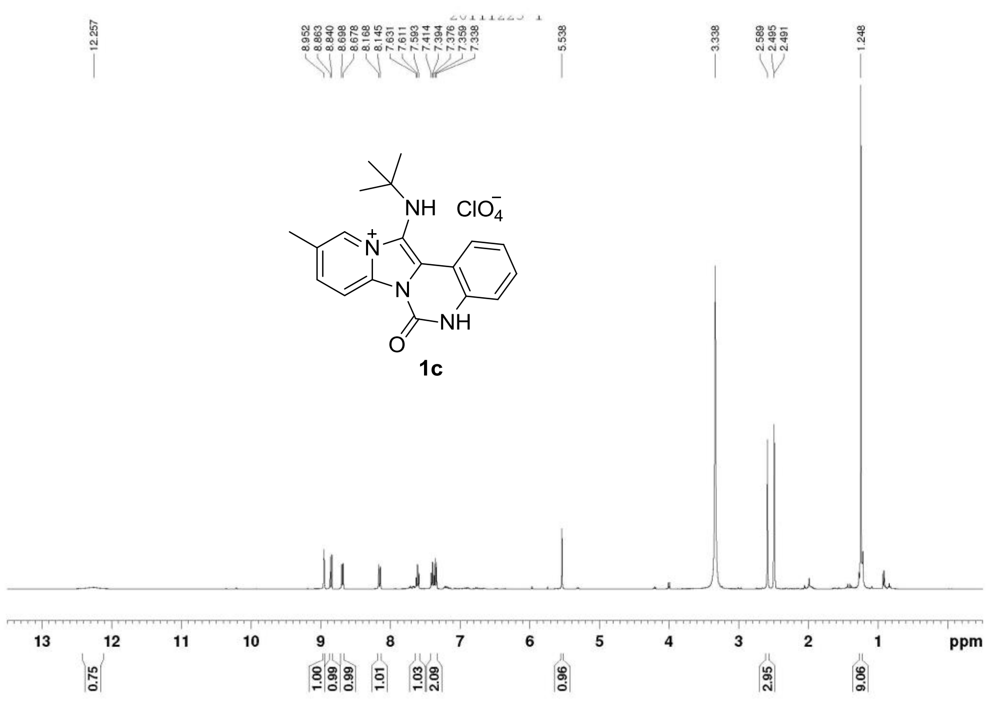

12.257
8.952
8.863
8.840
8.698
8.678
8.168
8.145
7.631
7.611
7.593
7.414
7.394
7.376
7.359
7.338
5.538
3.338
2.589
2.495
2.491
1.248
NH ClO4−
N+
N
NH
O
1c
ppm
0.75
1.00
0.99
0.99
1.01
1.03
2.09
0.96
2.95
9.06


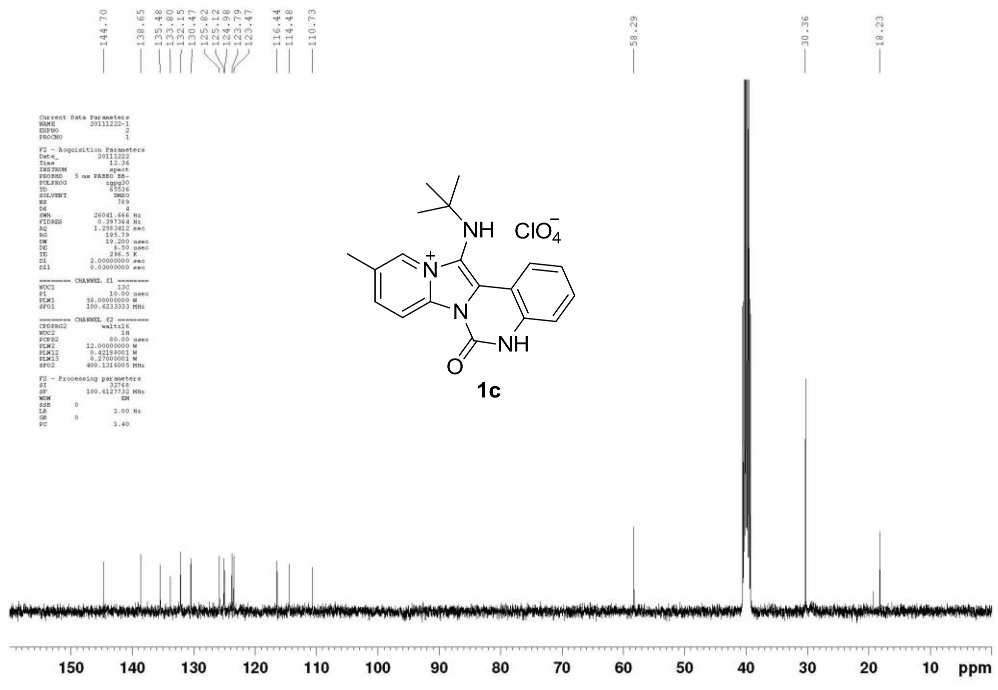

144.70
138.65
135.48
133.80
132.15
130.47
58.29
30.36
18.23
116.44
114.48
110.73
NH ClO4−
N+
N
NH
O
1c
ppm

**1d**

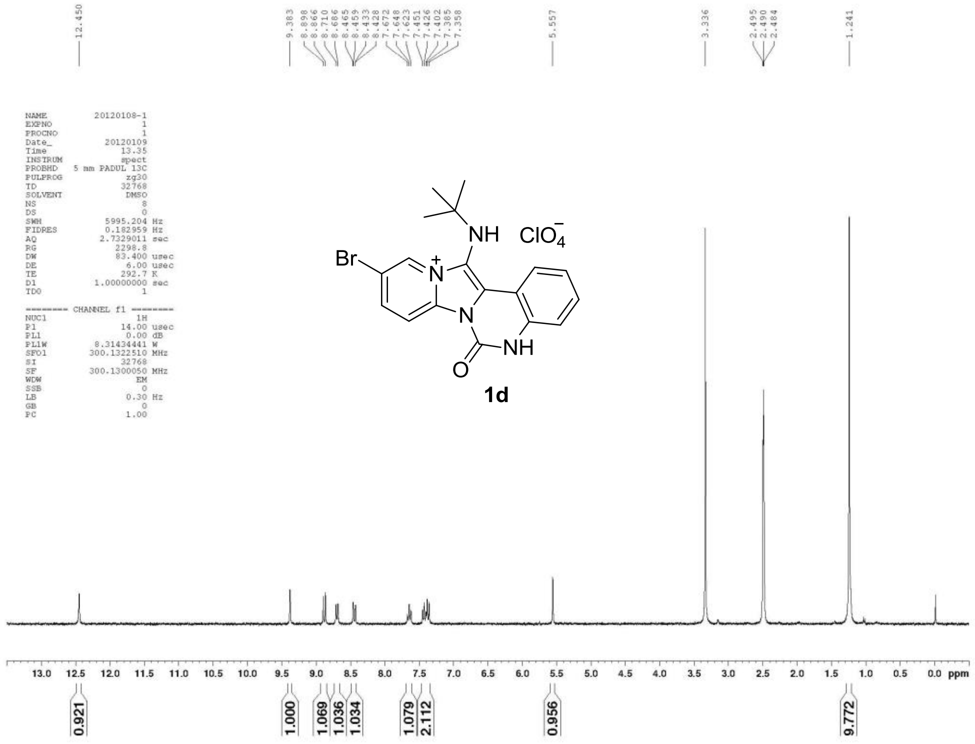
NAME 20120108-1
EXPNO 1
PROCNO 1
Date_ 20120109
Time 13.35
INSTRUM spect
PROBHD 5 mm PADUL 13C
PULPROG zg30
TD 32768
SOLVENT DMSO
NS 8
DS 0
SWH 5995.204 Hz
FIDRES 0.182959 Hz
AQ 2.7329011 sec
RG 2298.8
DW 83.400 usec
DE 6.00 usec
TE 292.7 K
D1 1.00000000 sec
TD0 1
======= CHANNEL f1 =======
NUC1 1H
P1 14.00 usec
PL1 0.00 dB
PL1W 8.31434441 W
SFO1 300.1322510 MHz
SI 32768
SF 300.1300050 MHz
WDW EM
SSB 0
LB 0.30 Hz
GB 0
PC 1.00
Br
NH
ClO4−
N+
N
NH
O
1d
12.450
9.383 8.898 8.866 8.710 8.686 8.465 8.459 8.433 8.428 7.672 7.648 7.623 7.451 7.426 7.402 7.385 7.358
5.557
3.336
2.495 2.490 2.484
1.241
0.921 1.000 1.069 1.036 1.034 1.079 2.112 0.956 9.772
ppm

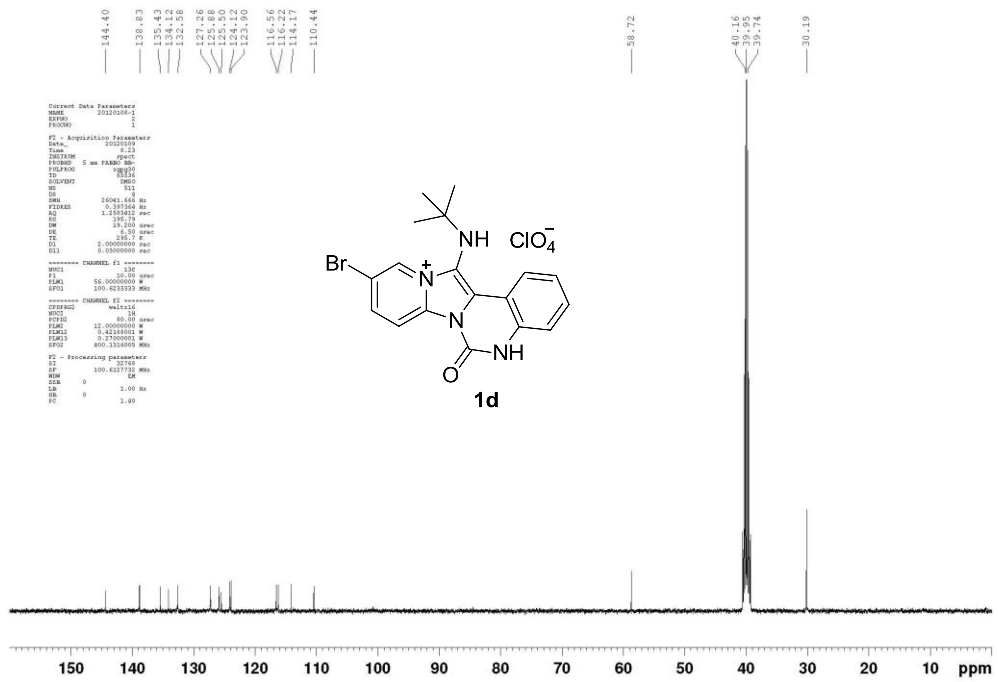


1e

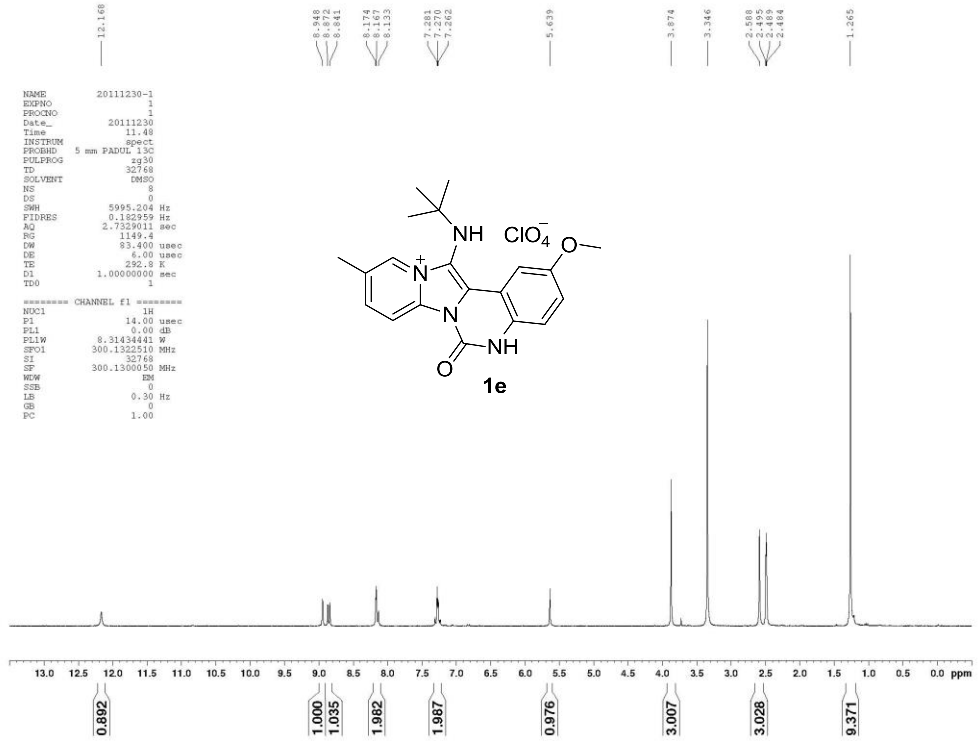

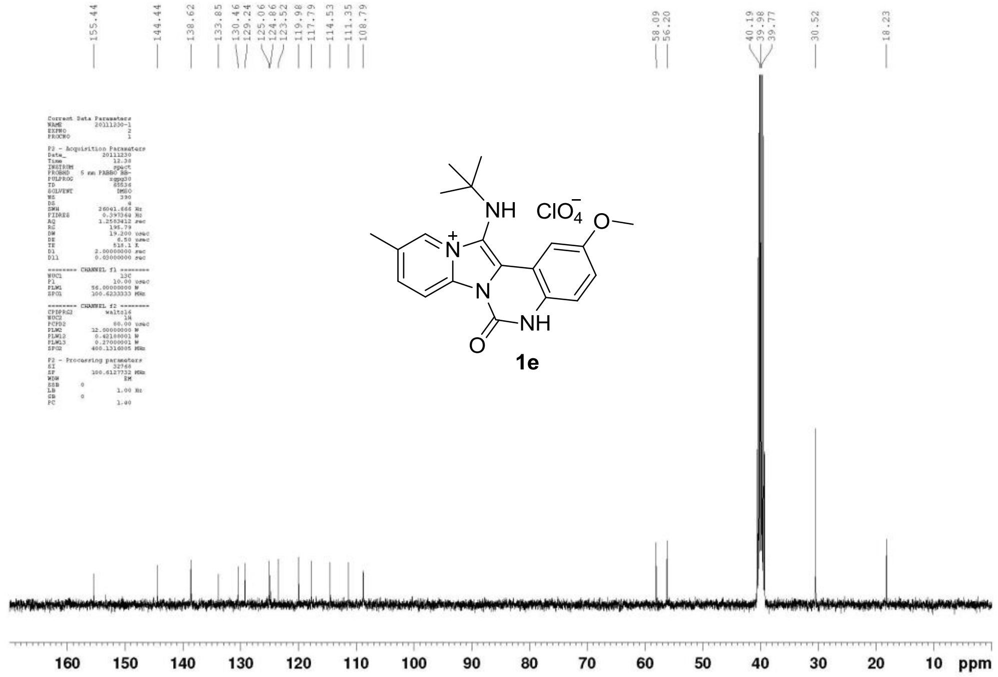
155.44
144.44
138.62
133.85
130.46
129.24
125.06
124.86
123.52
119.98
117.79
114.53
111.35
108.79
58.09
56.20
40.19
39.98
39.77
30.52
18.23
NH
$ClO_4^-$
1e
160 150 140 130 120 110 100 90 80 70 60 50 40 30 20 10 ppm

**1f**

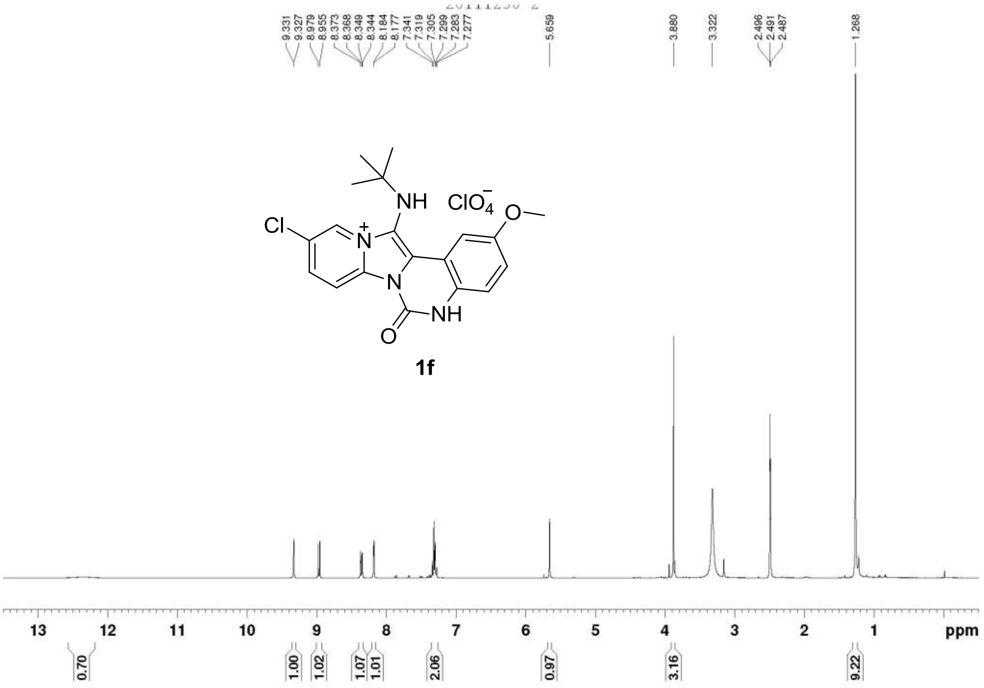
9.331
9.327
8.979
8.955
8.373
8.368
8.349
8.344
8.184
8.177
7.341
7.319
7.305
7.299
7.283
7.277
5.659
3.880
3.322
2.496
2.491
2.487
1.268
NH
$ClO_4^-$
Cl
1f
13 12 11 10 9 8 7 6 5 4 3 2 1 ppm
0.70
1.00
1.02
1.07
1.01
2.06
0.97
3.16
9.22

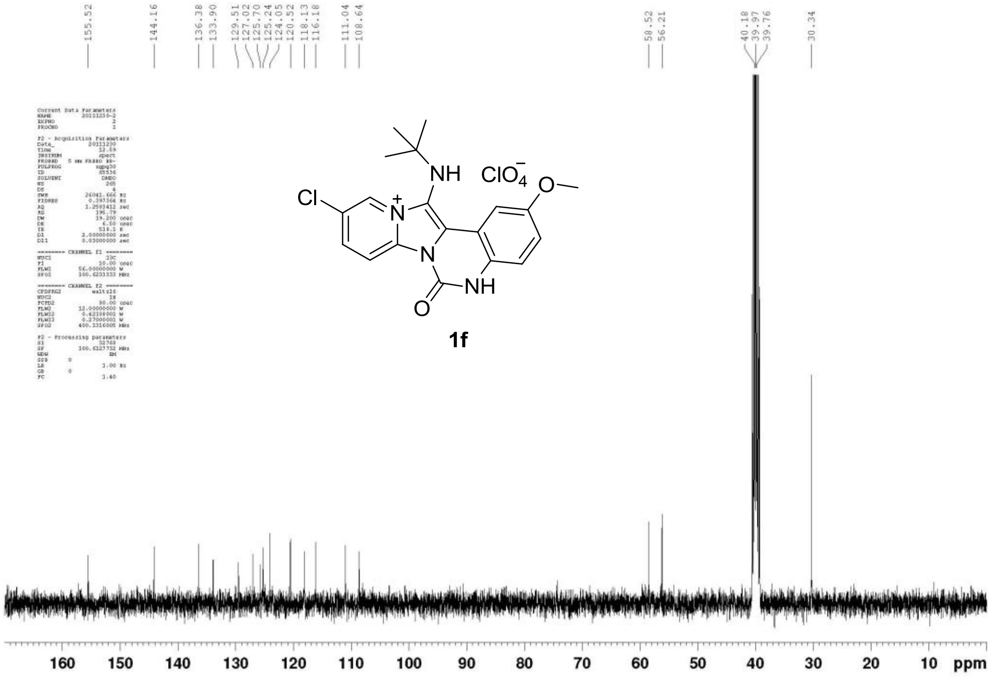
155.52
144.16
136.38
133.90
129.51
127.02
125.70
125.24
124.05
120.52
118.13
116.18
111.04
108.64
58.52
56.21
40.18
39.97
39.76
30.34
NH
ClO4−
Cl
N+
N
O
NH
O
1f
160 150 140 130 120 110 100 90 80 70 60 50 40 30 20 10 ppm

**1g**

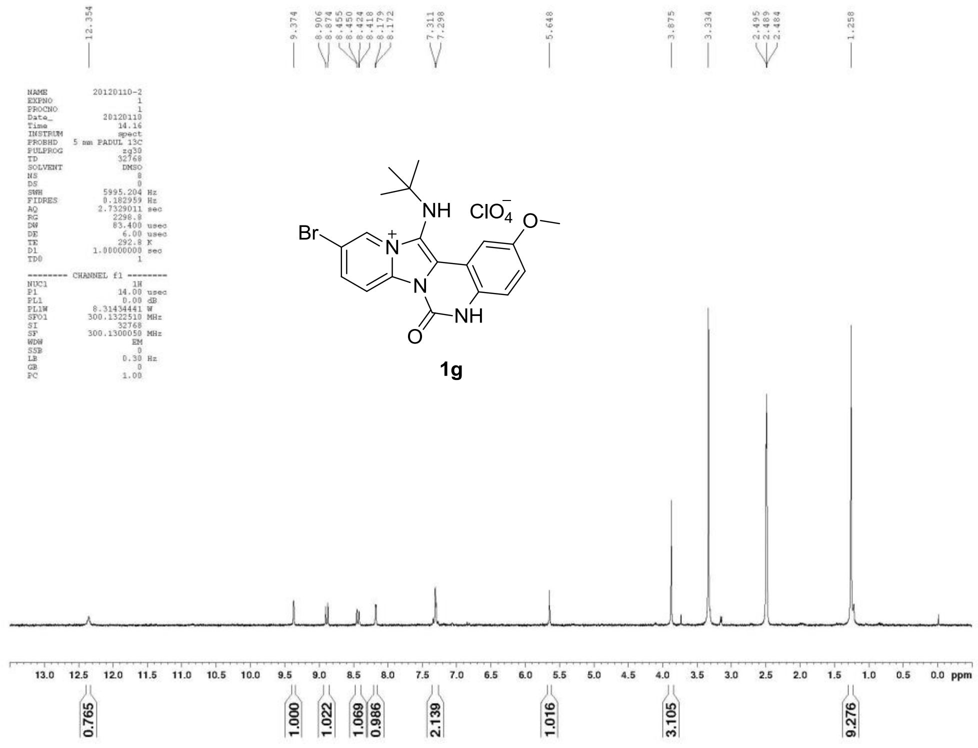

12.354
9.374
8.906
8.874
8.455
8.450
8.424
8.418
8.179
8.172
7.311
7.298
5.648
3.875
3.334
2.495
2.489
2.484
1.258
NAME 20120110-2
SOLVENT DMSO
NH
ClO4−
Br
N+
N
NH
O
1g
13.0 12.5 12.0 11.5 11.0 10.5 10.0 9.5 9.0 8.5 8.0 7.5 7.0 6.5 6.0 5.5 5.0 4.5 4.0 3.5 3.0 2.5 2.0 1.5 1.0 0.5 0.0 ppm
0.765
1.000
1.022
1.069
0.986
2.139
1.016
3.105
9.276


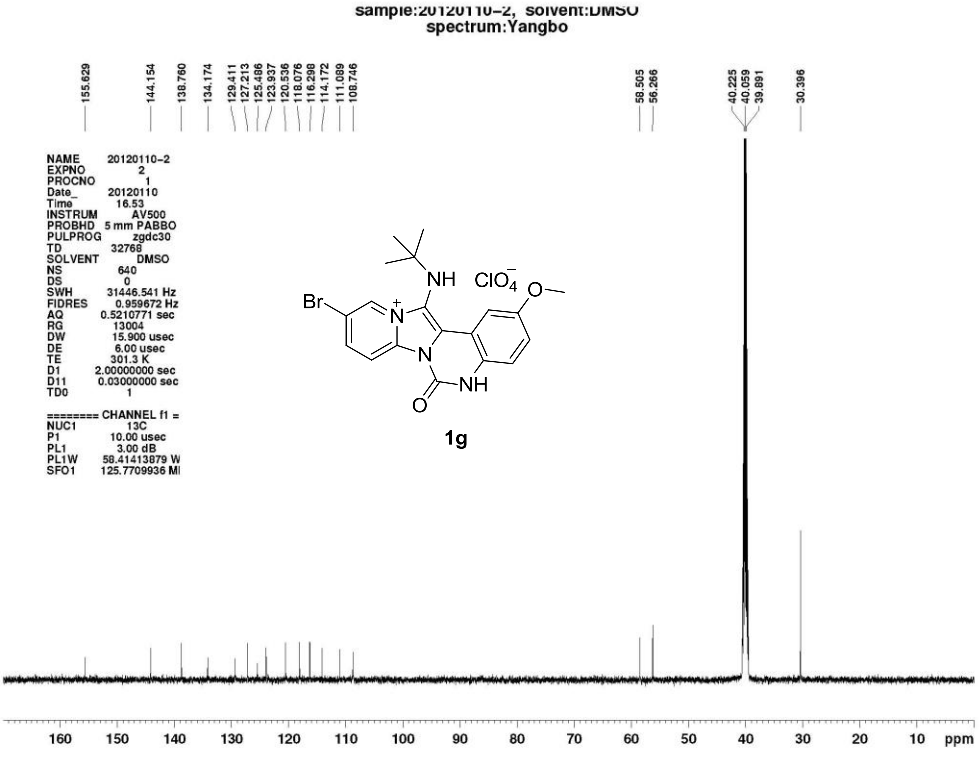

sample:20120110-2, solvent:DMSO
spectrum:Yangbo
155.629
144.154
138.760
134.174
129.411
127.213
125.486
123.937
120.536
118.076
116.298
114.172
111.089
108.746
58.505
56.266
40.225
40.059
39.891
30.396
NAME 20120110-2
EXPNO 2
PROCNO 1
Date_ 20120110
Time 16.53
INSTRUM AV500
PROBHD 5 mm PABBO
PULPROG zgdc30
TD 32768
SOLVENT DMSO
NS 640
DS 0
SWH 31446.541 Hz
FIDRES 0.959672 Hz
AQ 0.5210771 sec
RG 13004
DW 15.900 usec
DE 6.00 usec
TE 301.3 K
D1 2.00000000 sec
D11 0.03000000 sec
TD0 1
======== CHANNEL f1 =
NUC1 13C
P1 10.00 usec
PL1 3.00 dB
PL1W 58.41413879 W
SFO1 125.7709936 MI
NH
ClO4−
Br
N+
N
NH
O
1g
160 150 140 130 120 110 100 90 80 70 60 50 40 30 20 10 ppm

**1h**

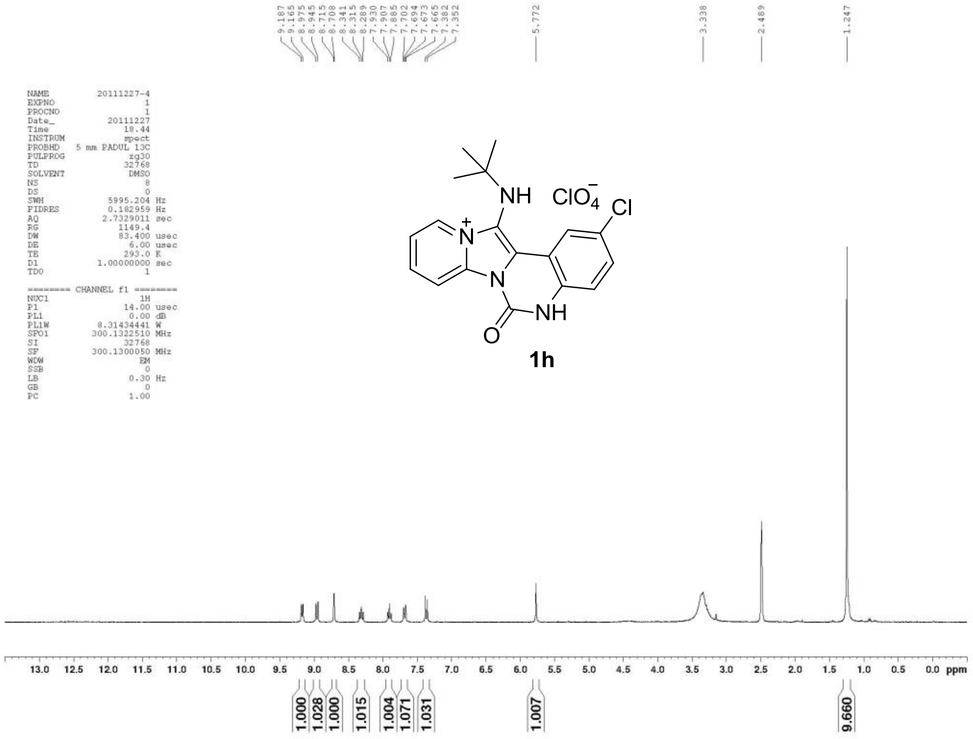
NH
$ClO_4^-$
Cl
N
NH
O
1h
ppm

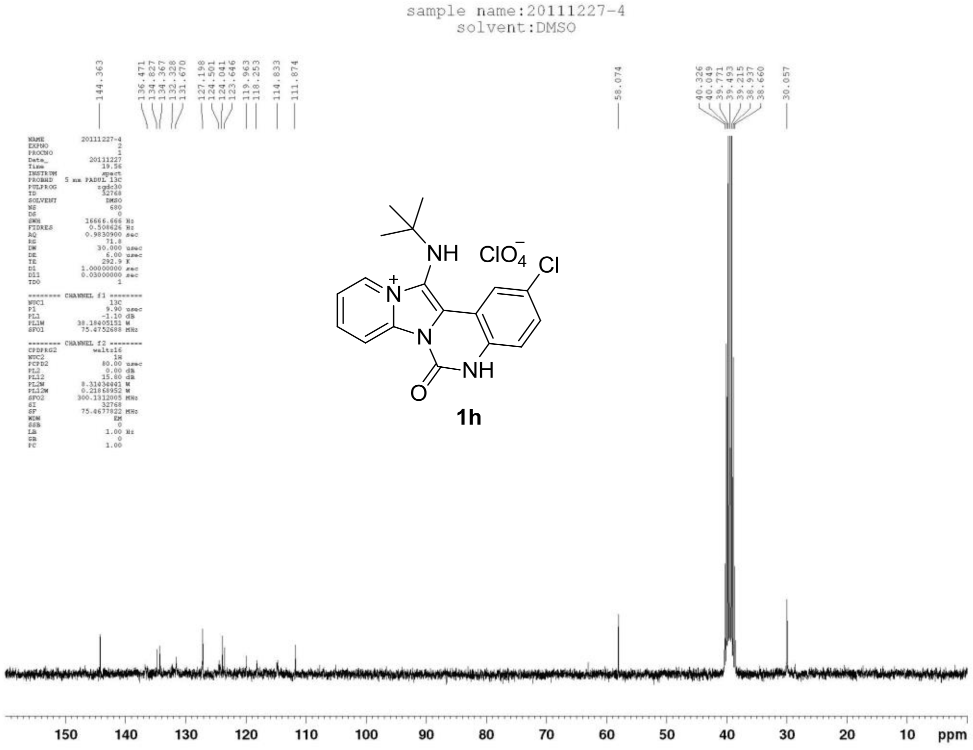
sample name:20111227-4
solvent:DMSO
NH
$ClO_4^-$
Cl
N
NH
O
1h
ppm

**1i**

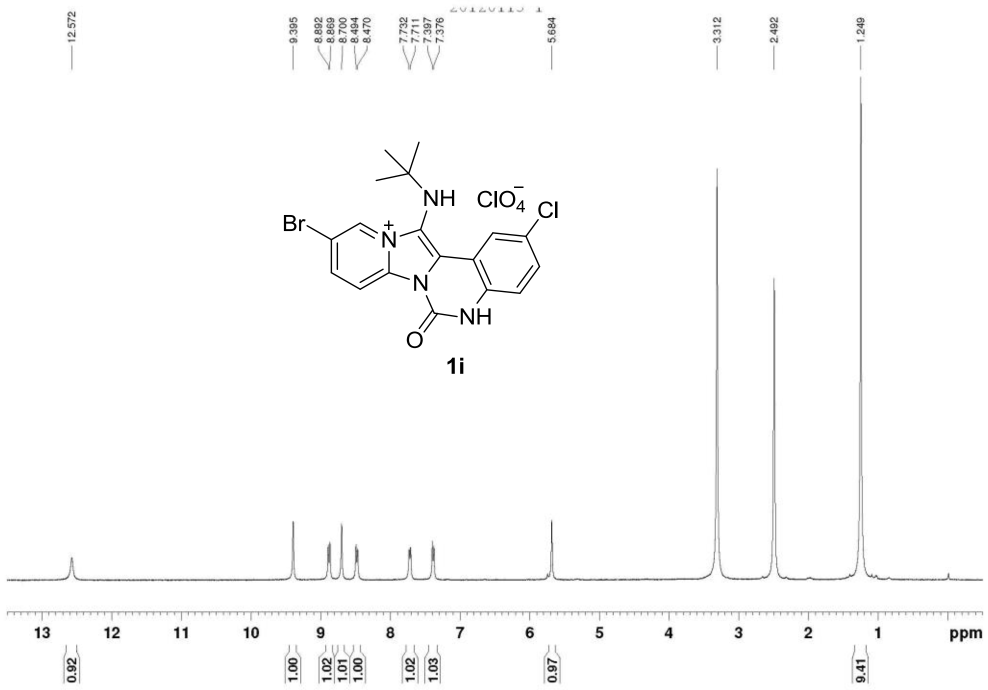

12.572
9.395
8.892
8.869
8.700
8.494
8.470
7.732
7.711
7.397
7.376
5.684
3.312
2.492
1.249
NH
ClO4
Cl
Br
N
N
NH
O
1i
13
12
11
10
9
8
7
6
5
4
3
2
1
ppm
0.92
1.00
1.02
1.01
1.00
1.02
1.03
0.97
9.41

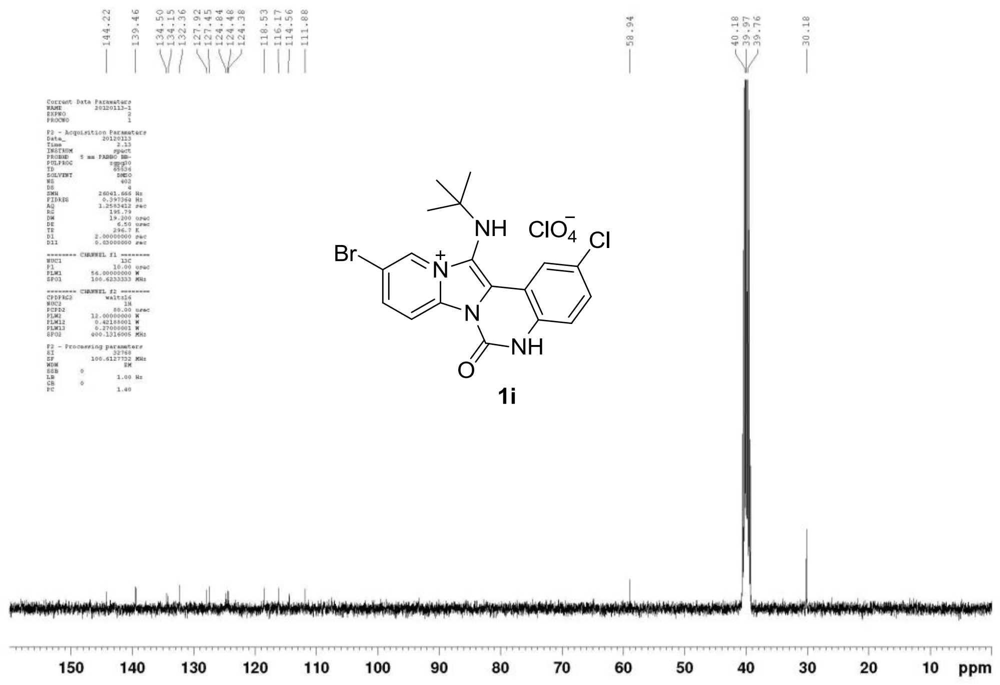

**1j**

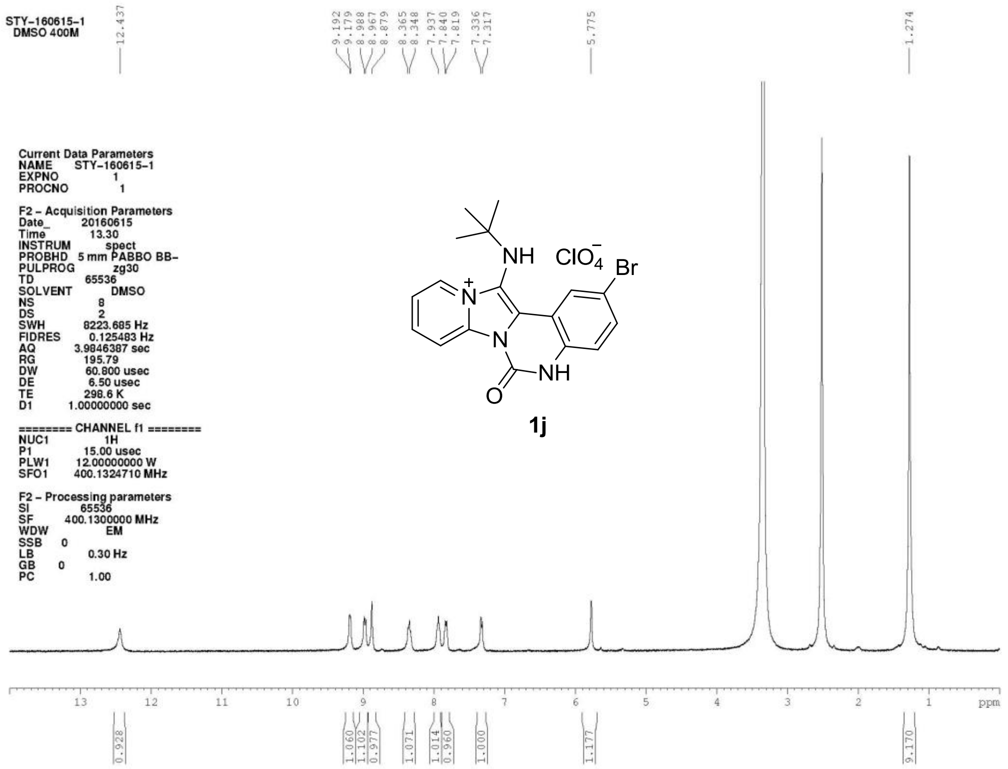
STY-160615-1
DMSO 400M
12.437
9.192 9.179 8.988 8.967 8.879 8.365 8.348 7.937 7.840 7.819 7.336 7.317
5.775
1.274
Current Data Parameters
NAME STY-160615-1
EXPNO 1
PROCNO 1
F2 - Acquisition Parameters
Date_ 20160615
Time 13.30
INSTRUM spect
PROBHD 5 mm PABBO BB-
PULPROG zg30
TD 65536
SOLVENT DMSO
NS 8
DS 2
SWH 8223.685 Hz
FIDRES 0.125483 Hz
AQ 3.9846387 sec
RG 195.79
DW 60.800 usec
DE 6.50 usec
TE 298.6 K
D1 1.00000000 sec
======== CHANNEL f1 ========
NUC1 1H
P1 15.00 usec
PLW1 12.00000000 W
SFO1 400.1324710 MHz
F2 - Processing parameters
SI 65536
SF 400.1300000 MHz
WDW EM
SSB 0
LB 0.30 Hz
GB 0
PC 1.00
NH
$ClO_4^-$
Br
N
N
NH
O
1j
ppm
0.928 1.060 1.102 0.977 1.071 1.014 0.960 1.000 1.177 9.170

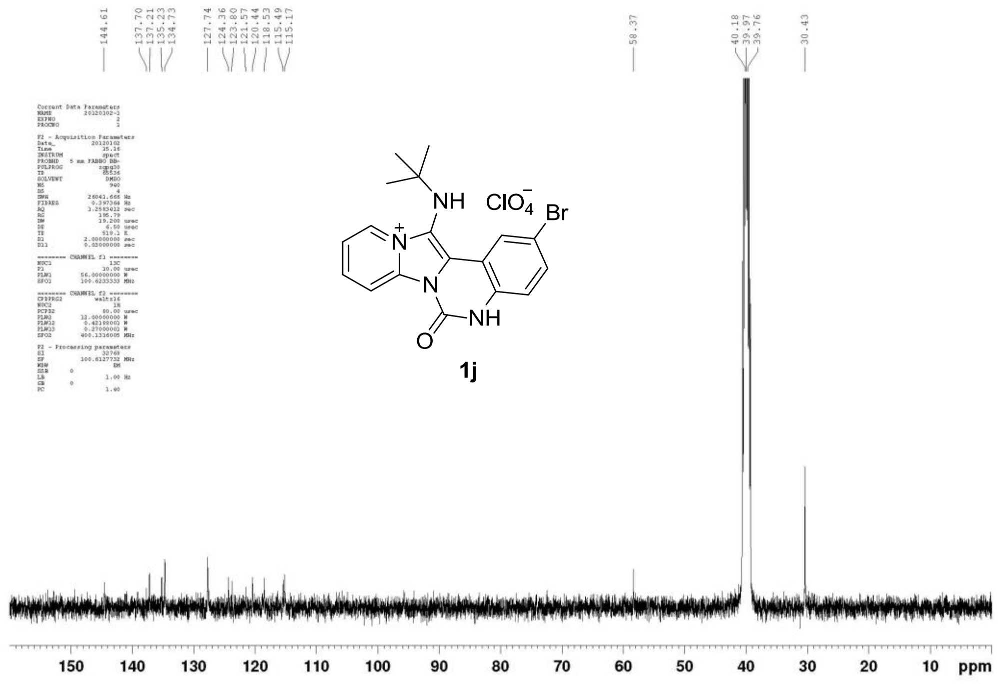
144.61
137.70 137.21 135.23 134.73
127.74 124.36 123.80 121.57 120.44 118.53 115.49 115.17
58.37
40.18 39.97 39.76
30.43
NH
$ClO_4^-$
Br
N
N
NH
O
1j
150 140 130 120 110 100 90 80 70 60 50 40 30 20 10 ppm

**1k**

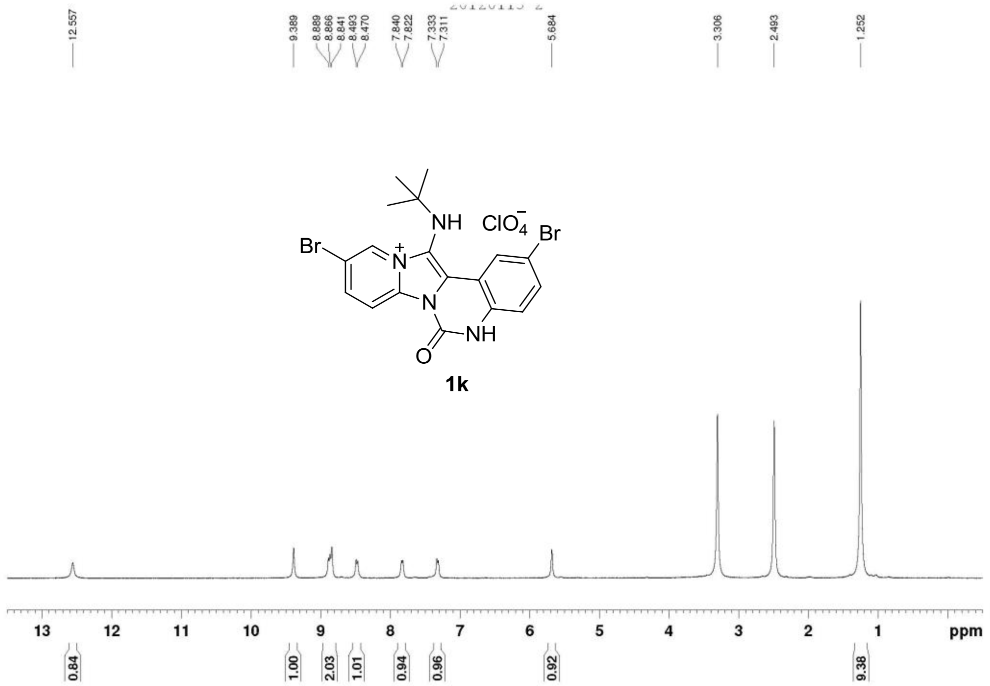
12.557
9.389
8.889
8.866
8.841
8.493
8.470
7.840
7.822
7.333
7.311
5.684
3.306
2.493
1.252
NH
ClO4−
Br
Br
N+
N
NH
O
1k
13 12 11 10 9 8 7 6 5 4 3 2 1 ppm
0.84
1.00
2.03
1.01
0.94
0.96
0.92
9.38

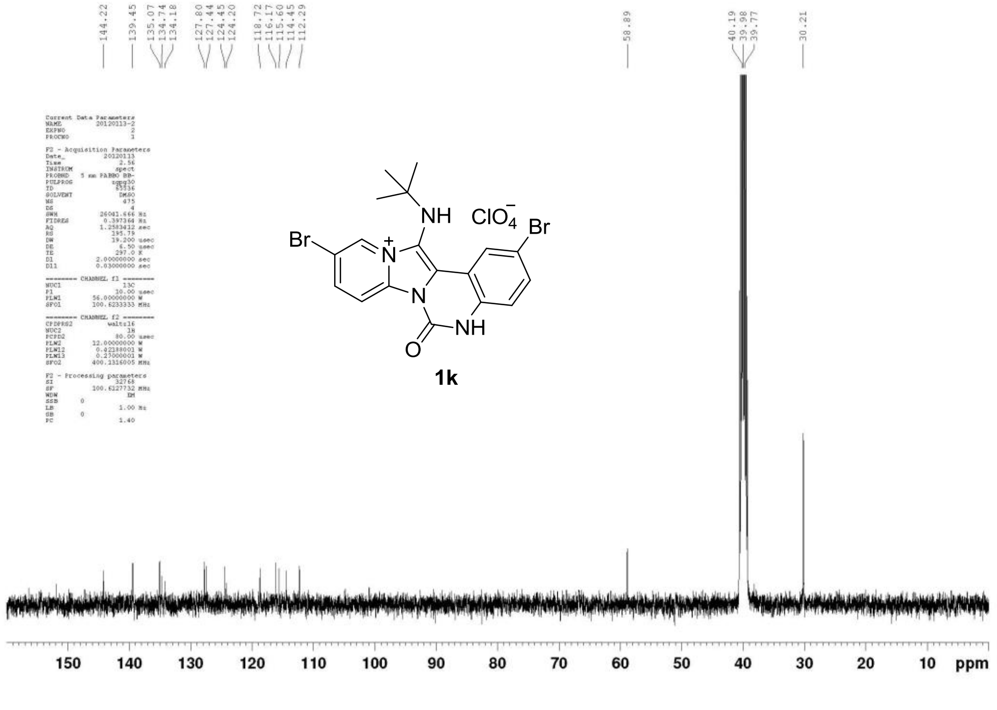
144.22
139.45
135.07
134.74
134.18
127.80
127.44
124.45
124.20
118.72
116.17
115.60
114.45
112.29
58.89
40.19
39.98
39.77
30.21
NH
ClO4−
Br
Br
N+
N
NH
O
1k
150 140 130 120 110 100 90 80 70 60 50 40 30 20 10 ppm

**1l**

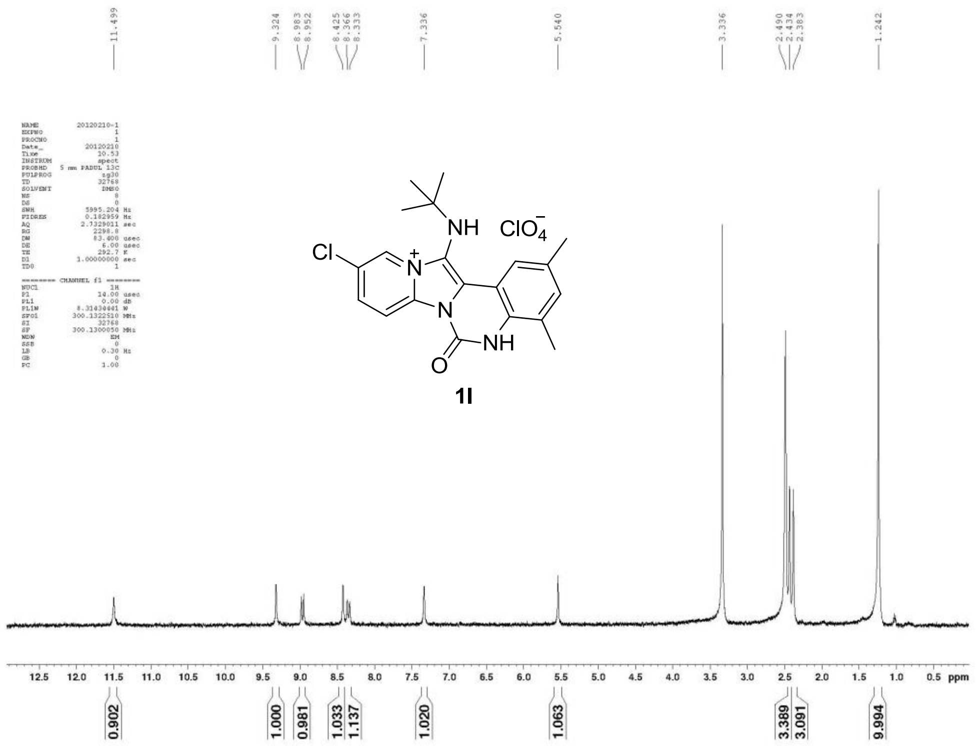

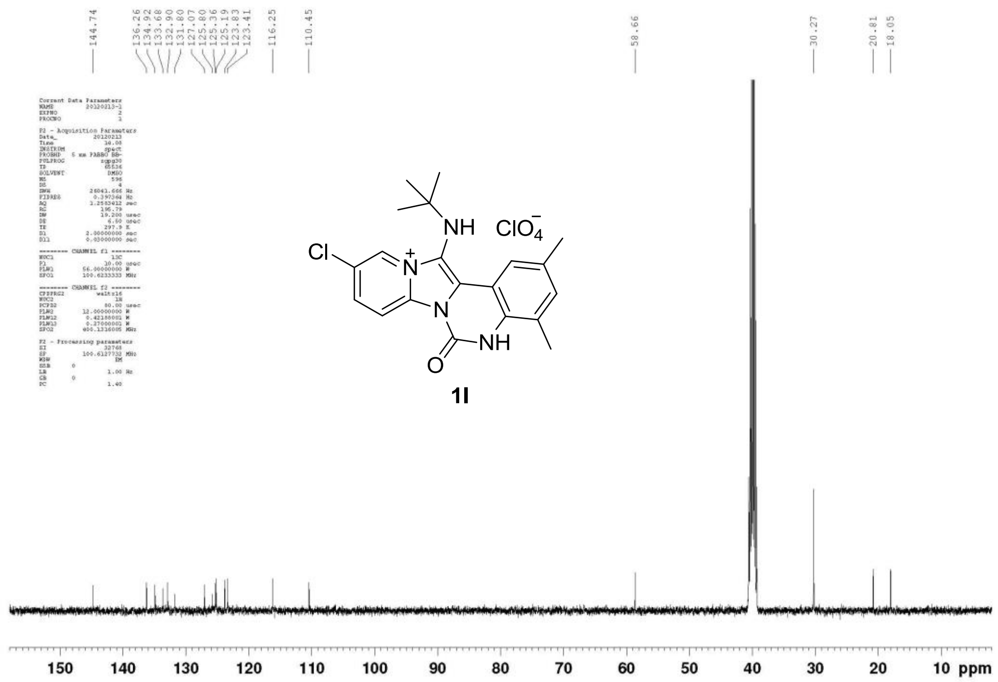

**1m**

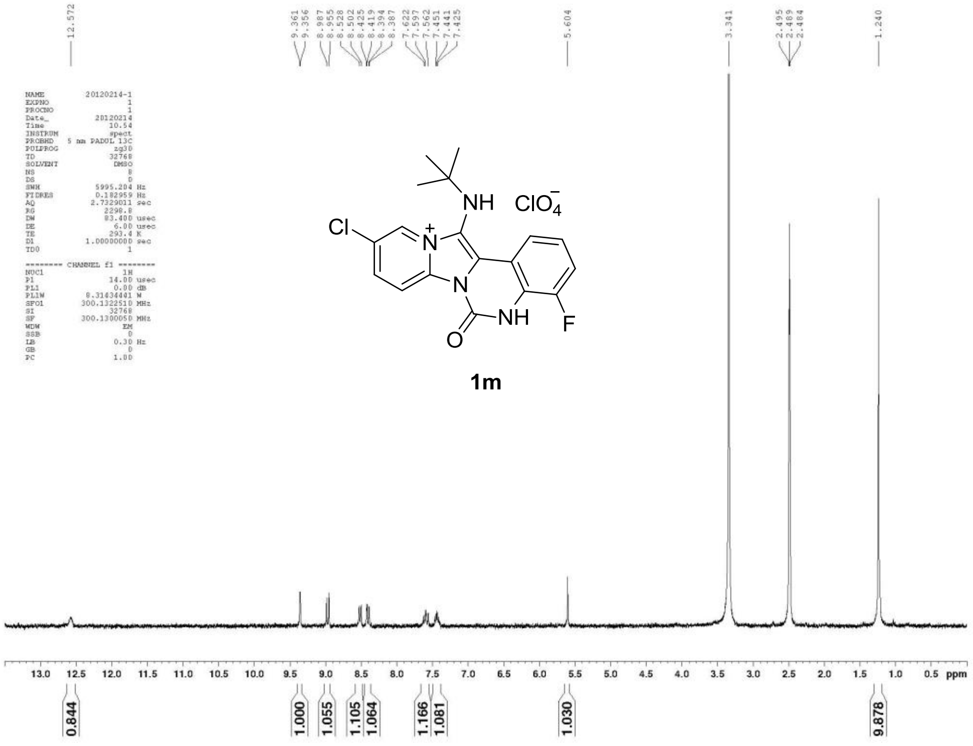

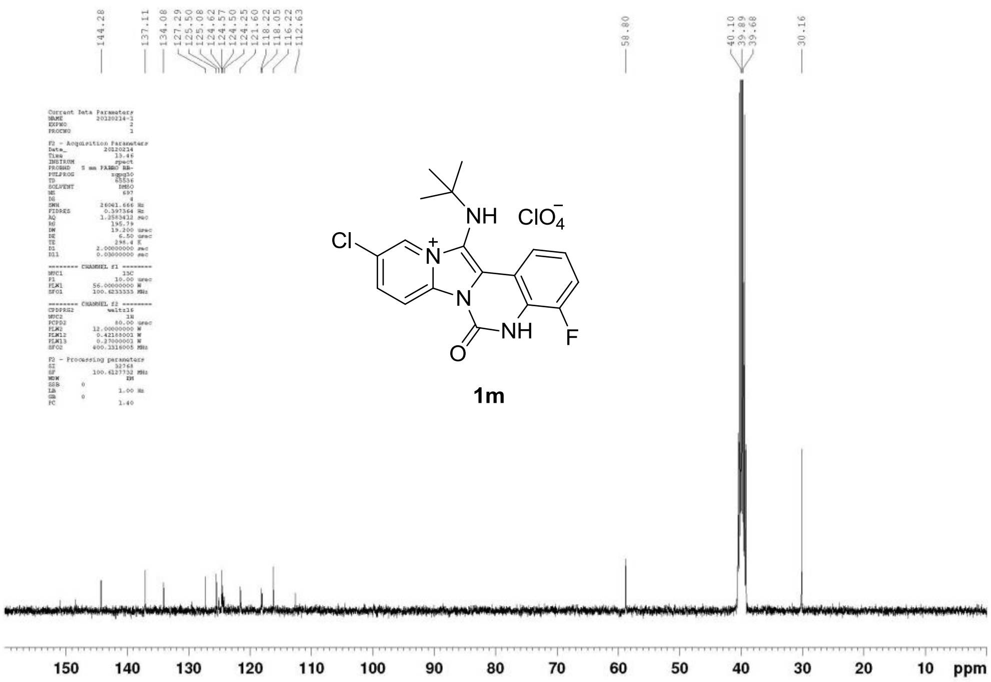


**1n**

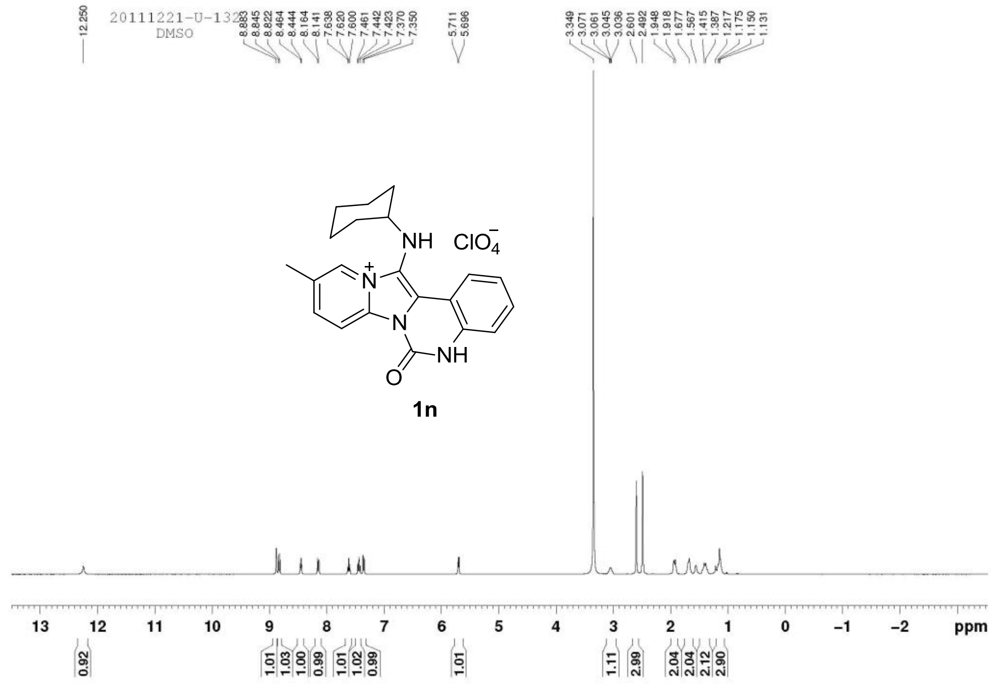

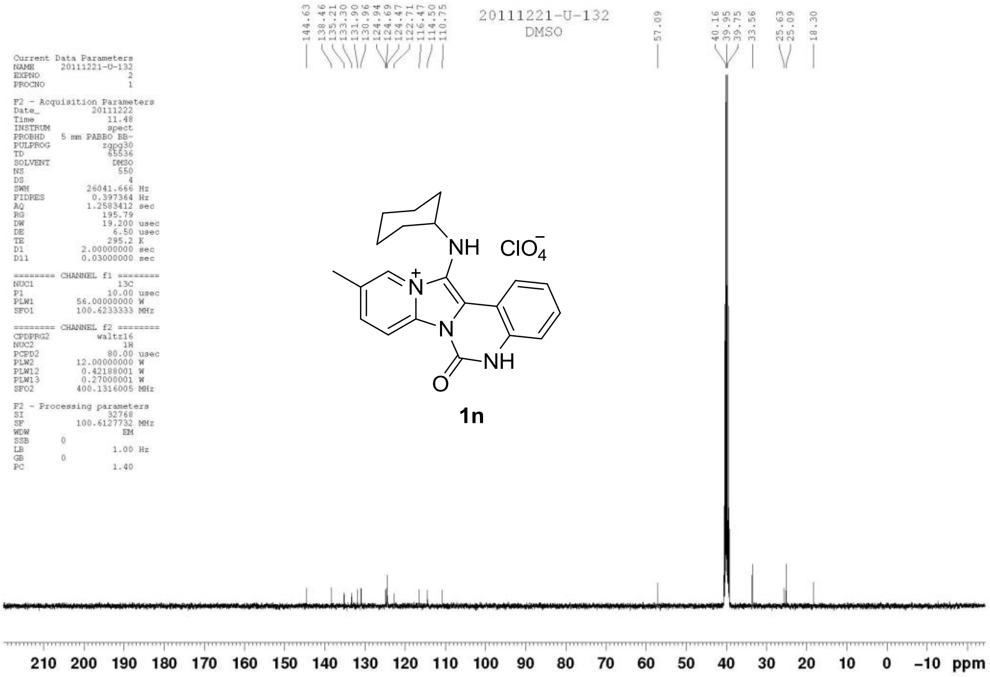
144.63
138.46
135.21
133.30
131.90
130.96
124.94
124.69
124.47
122.71
116.47
114.50
110.75
20111221-U-132
DMSO
57.09
40.16
39.95
39.75
33.56
25.63
25.09
18.30
NH
ClO4−
N+
N
NH
O
1n
210 200 190 180 170 160 150 140 130 120 110 100 90 80 70 60 50 40 30 20 10 0 −10 ppm

**1o**

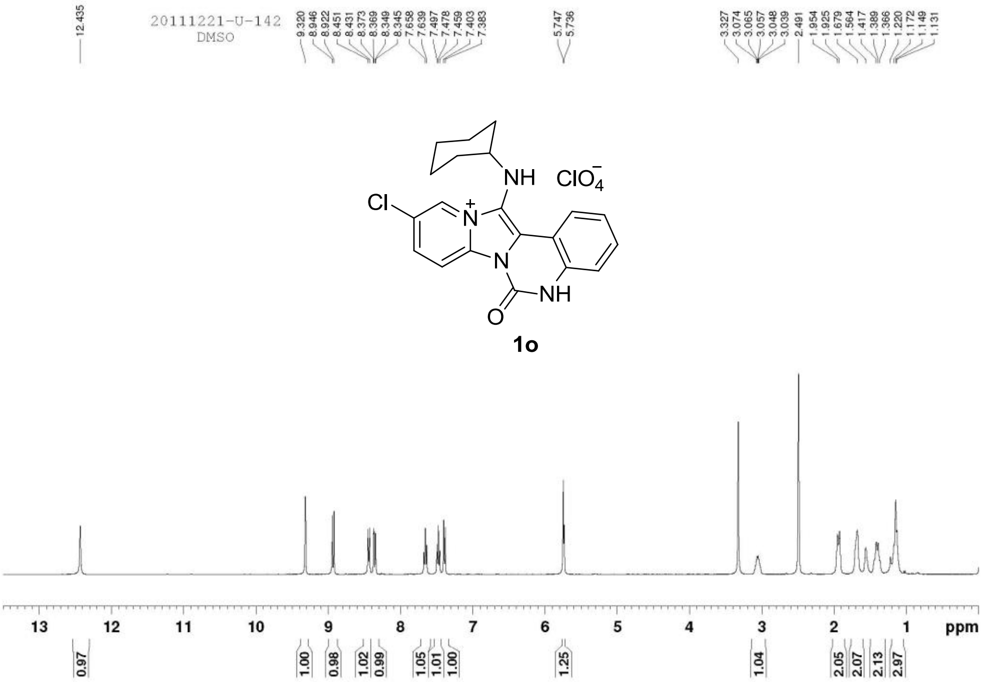
20111221-U-142
DMSO
NH
$ClO_4^-$
Cl
N
NH
O
1o
ppm

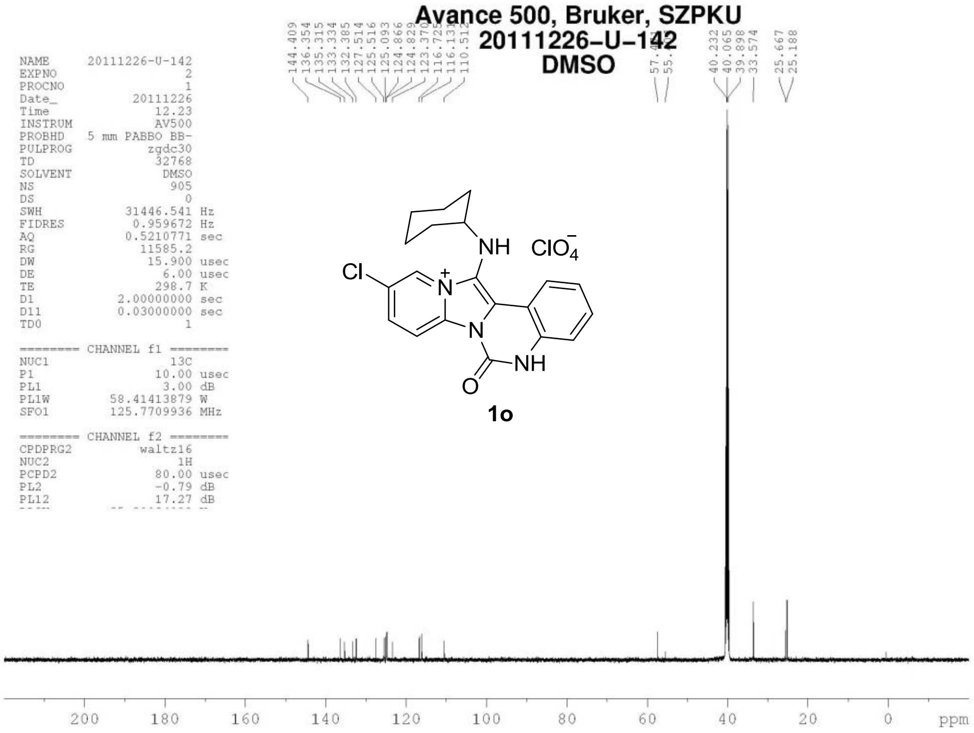
Avance 500, Bruker, SZPKU
20111226-U-142
DMSO
NH
$ClO_4^-$
Cl
N
NH
O
1o
ppm

**1p**

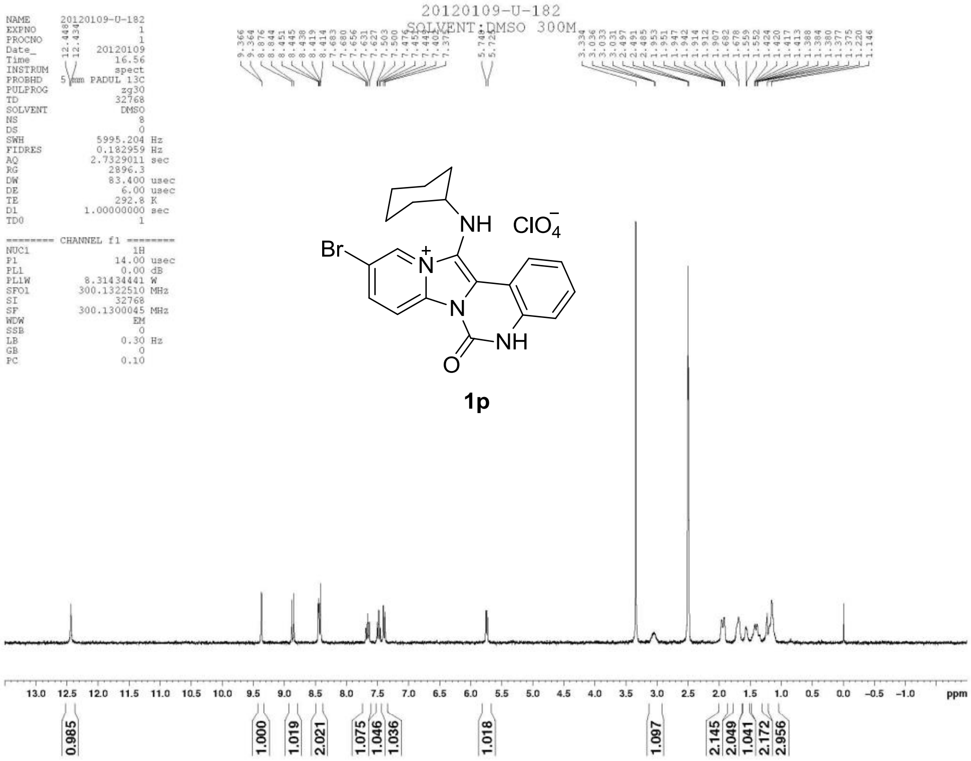
20120109-U-182
SOLVENT:DMSO 300M
NH ClO4−
Br
N+
N
NH
O
1p
ppm

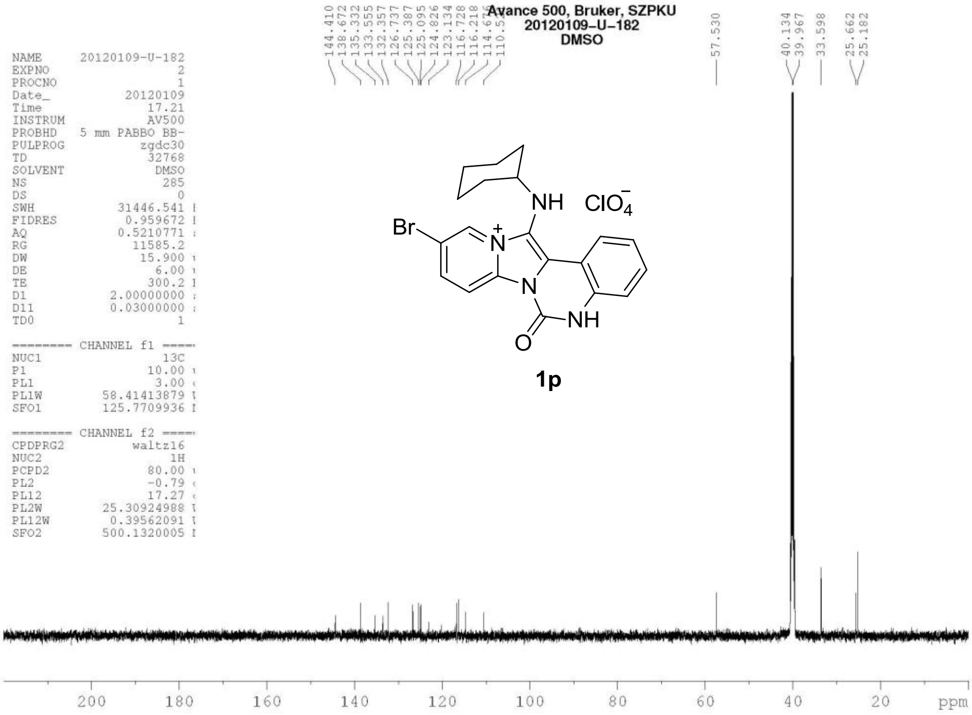
Avance 500, Bruker, SZPKU
20120109–U–182
DMSO
NH ClO4−
Br
N+
N
NH
O
1p
ppm

**1q**

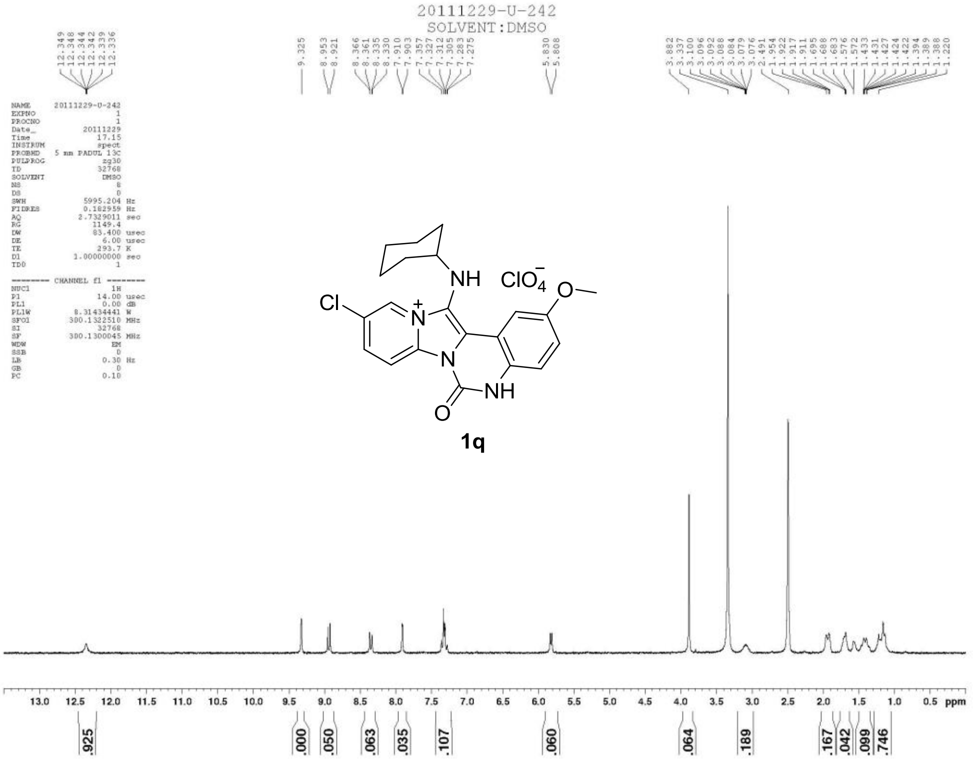

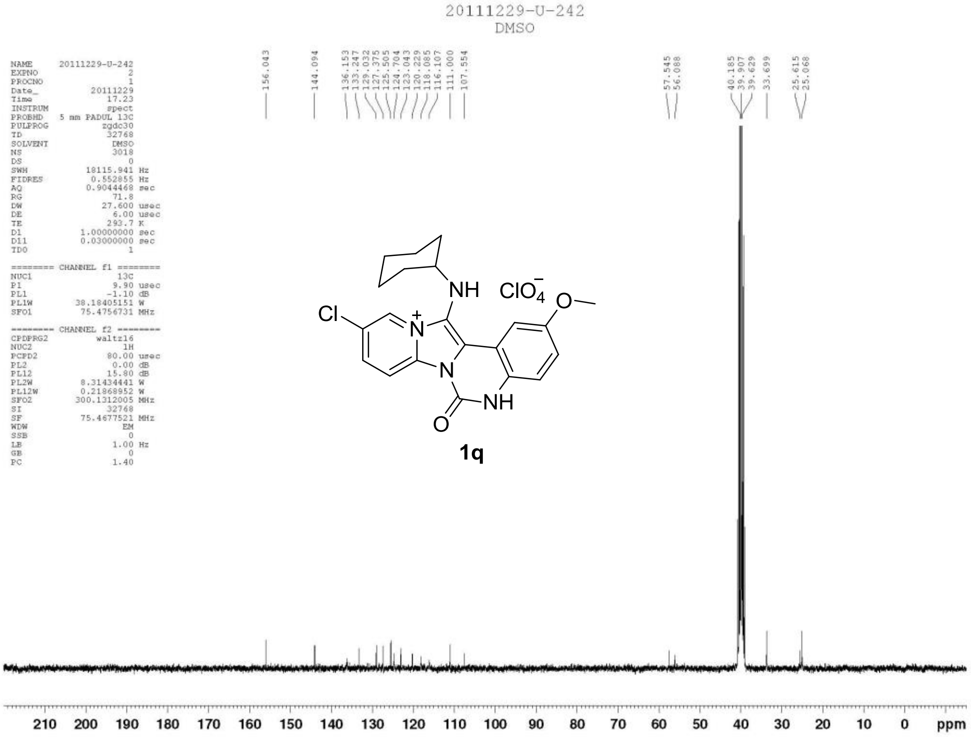

**1r**

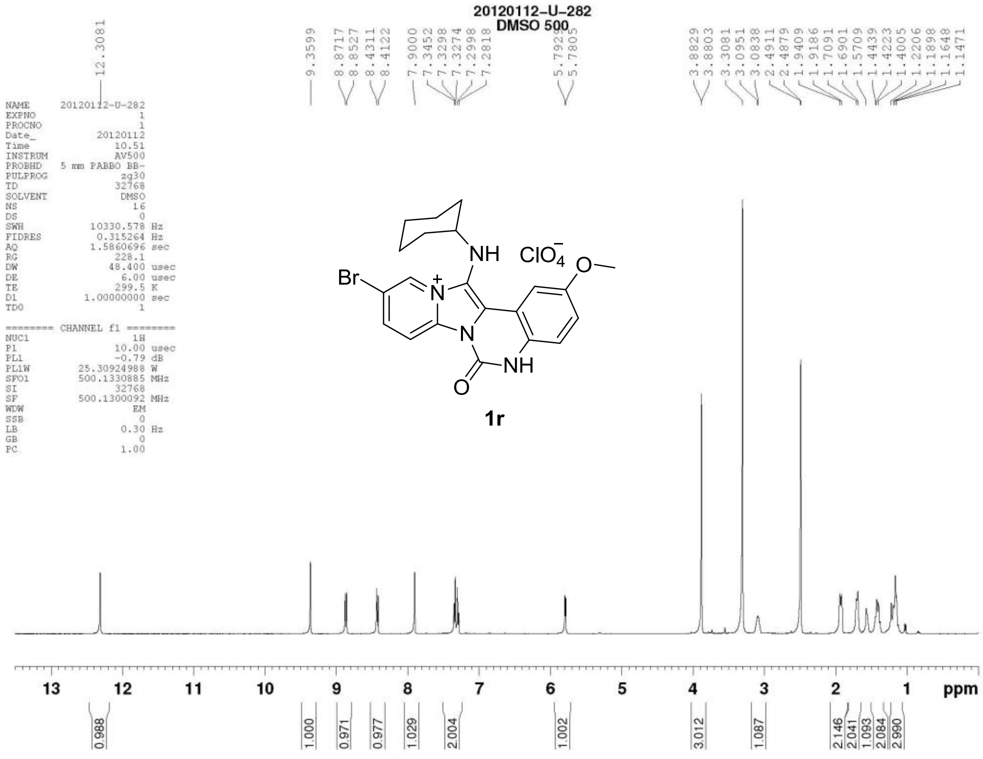
20120112-U-282
DMSO 500
1r
ppm

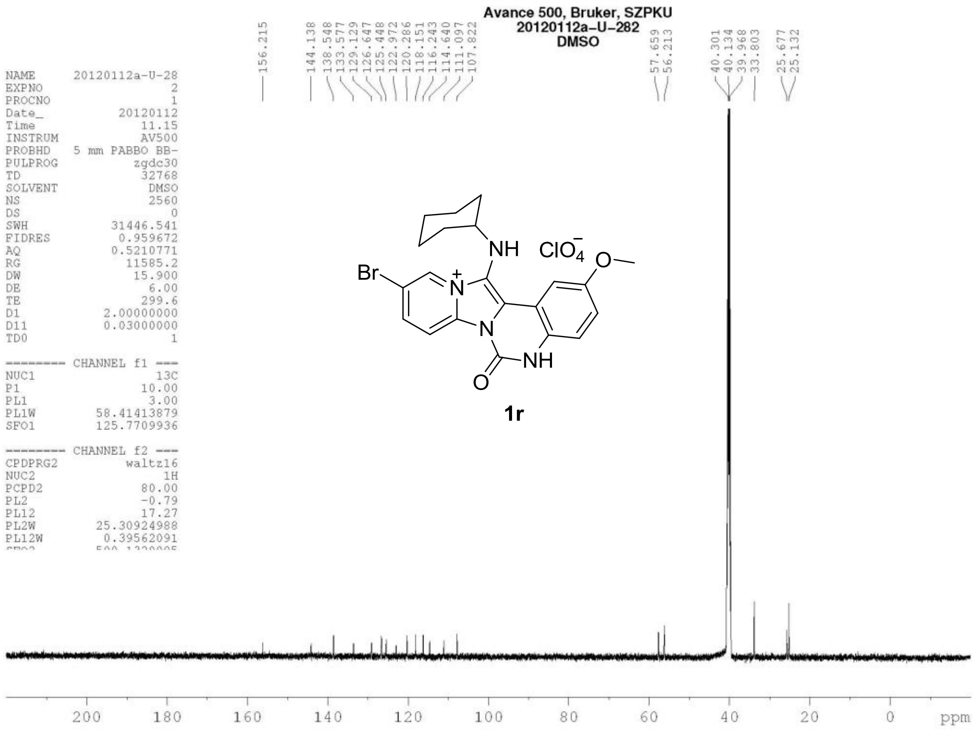
Avance 500, Bruker, SZPKU
20120112a-U-282
DMSO
1r
ppm

**1s**

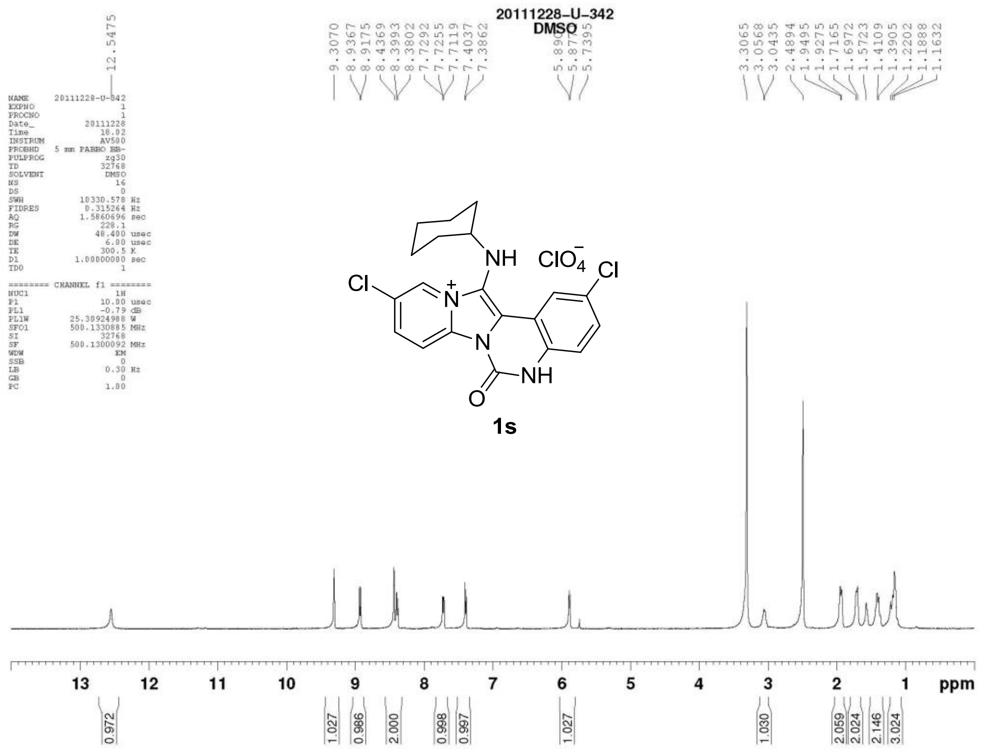
20111228–U–342
DMSO
12.5475
9.3070
8.9367
8.9175
8.4369
8.3993
8.3802
7.7292
7.7255
7.7119
7.4037
7.3862
5.7395
3.3065
3.0568
3.0435
2.4894
1.9495
1.9275
1.7165
1.6972
1.5723
1.4109
1.3905
1.2202
1.1888
1.1632
NH
ClO4−
Cl
Cl
N+
N
NH
O
1s
13 12 11 10 9 8 7 6 5 4 3 2 1 ppm
0.972
1.027
0.986
2.000
0.998
0.997
1.027
1.030
2.059
2.024
2.146
3.024

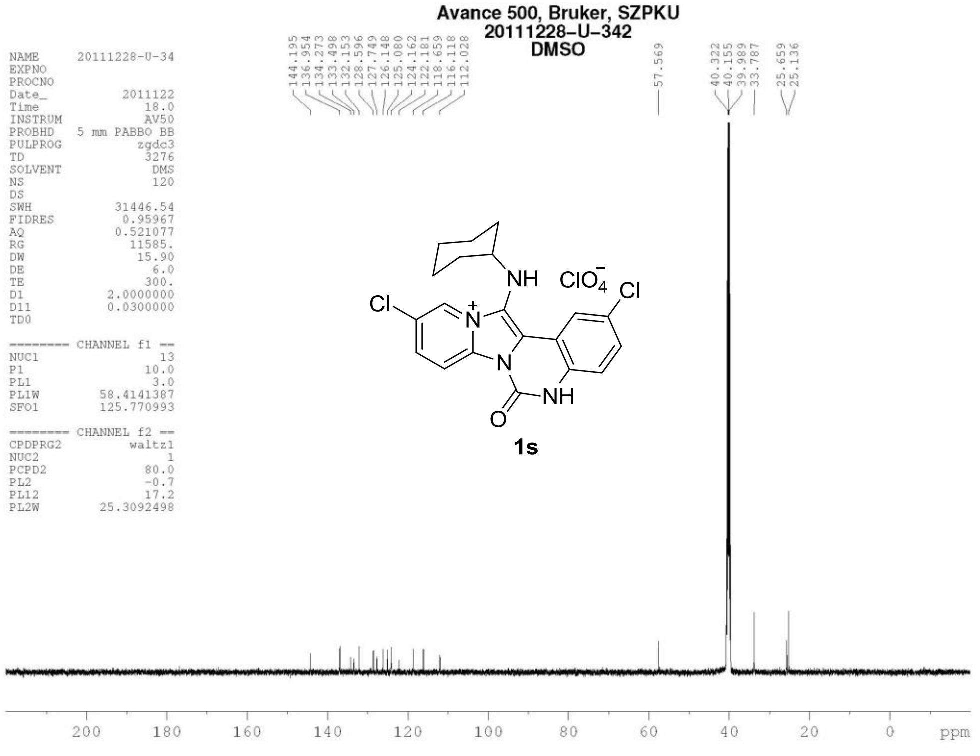
Avance 500, Bruker, SZPKU
20111228–U–342
DMSO
144.195
136.954
134.273
133.498
132.153
128.596
127.749
126.148
125.080
124.162
122.181
118.659
116.118
112.028
57.569
40.322
40.155
39.989
33.787
25.659
25.136
NH
ClO4−
Cl
Cl
N+
N
NH
O
1s
200 180 160 140 120 100 80 60 40 20 0 ppm

**1t**

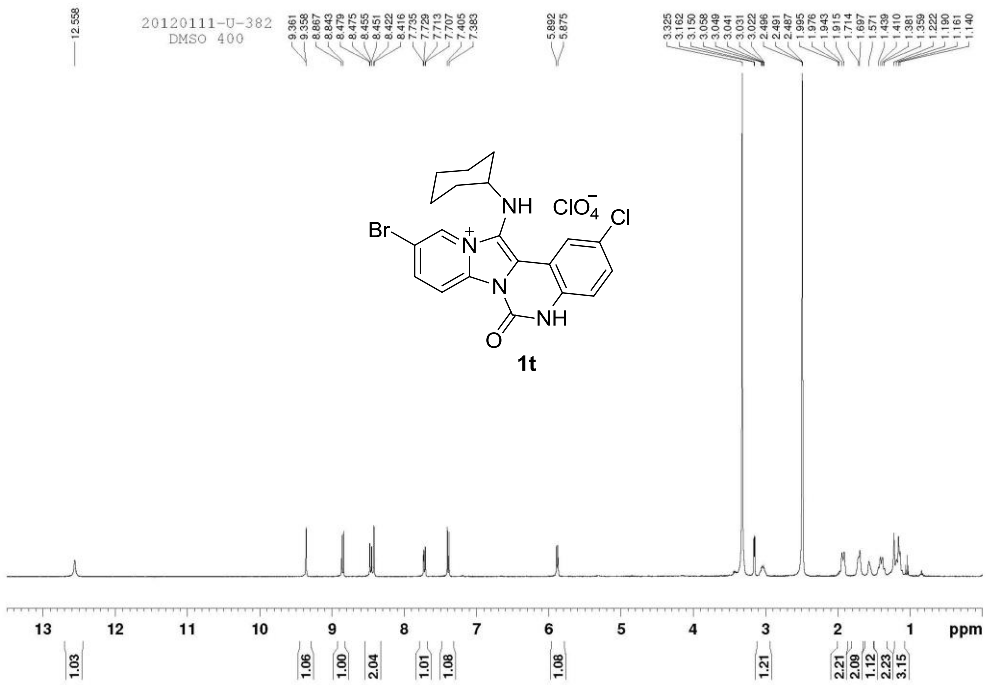

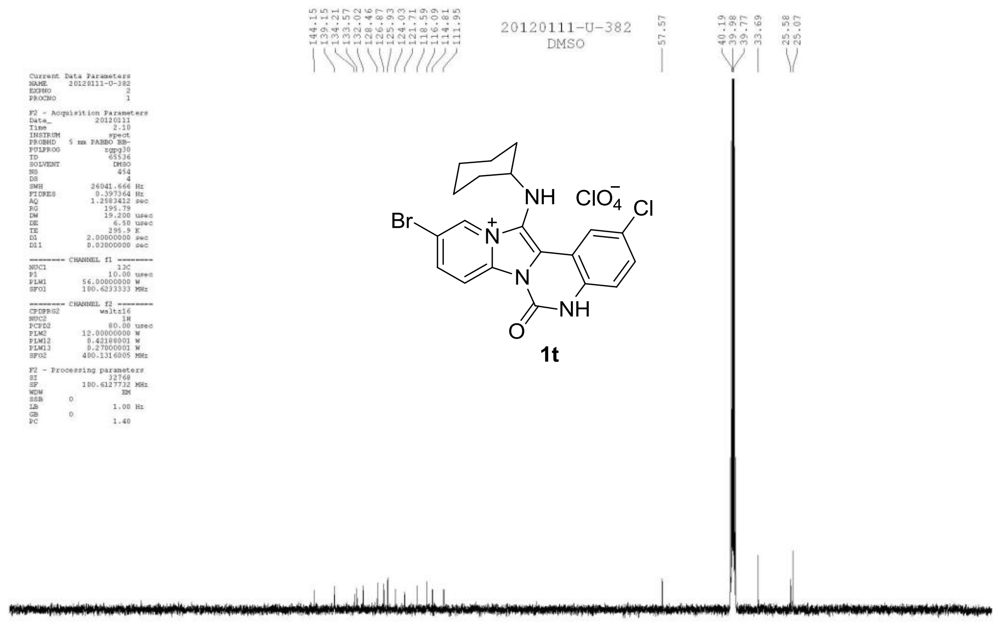

**1u**

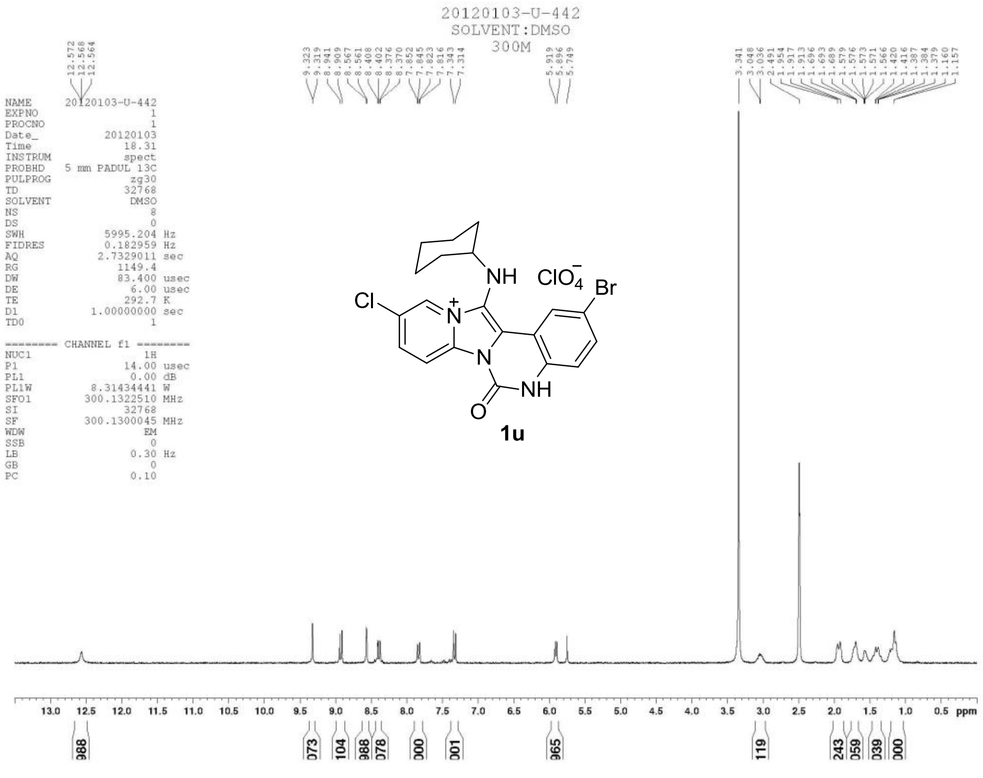

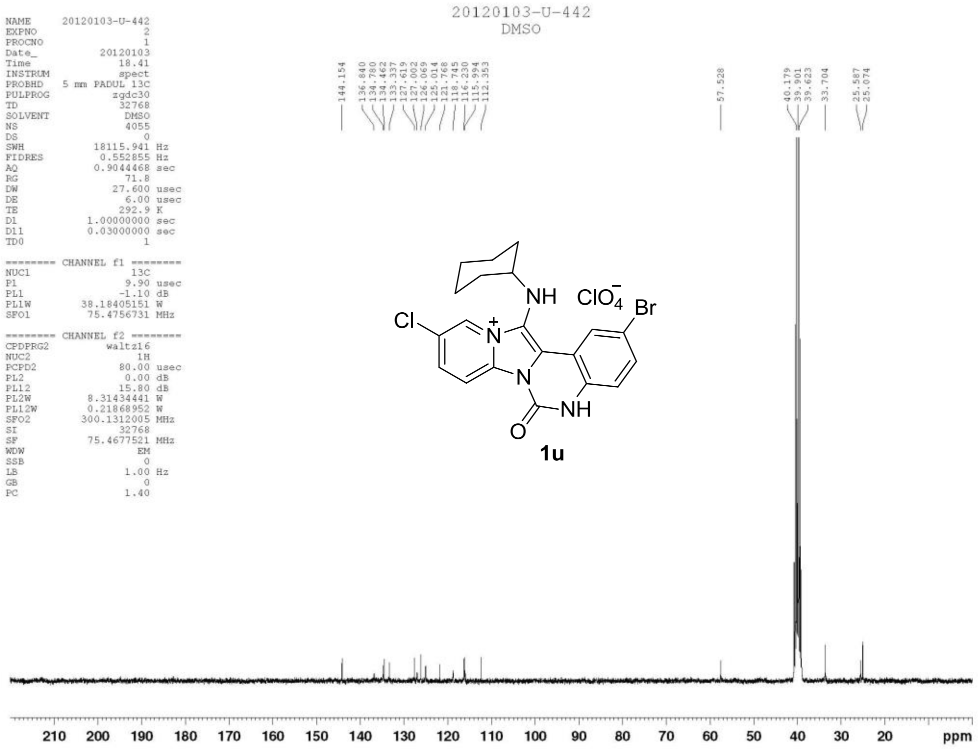


**1v**

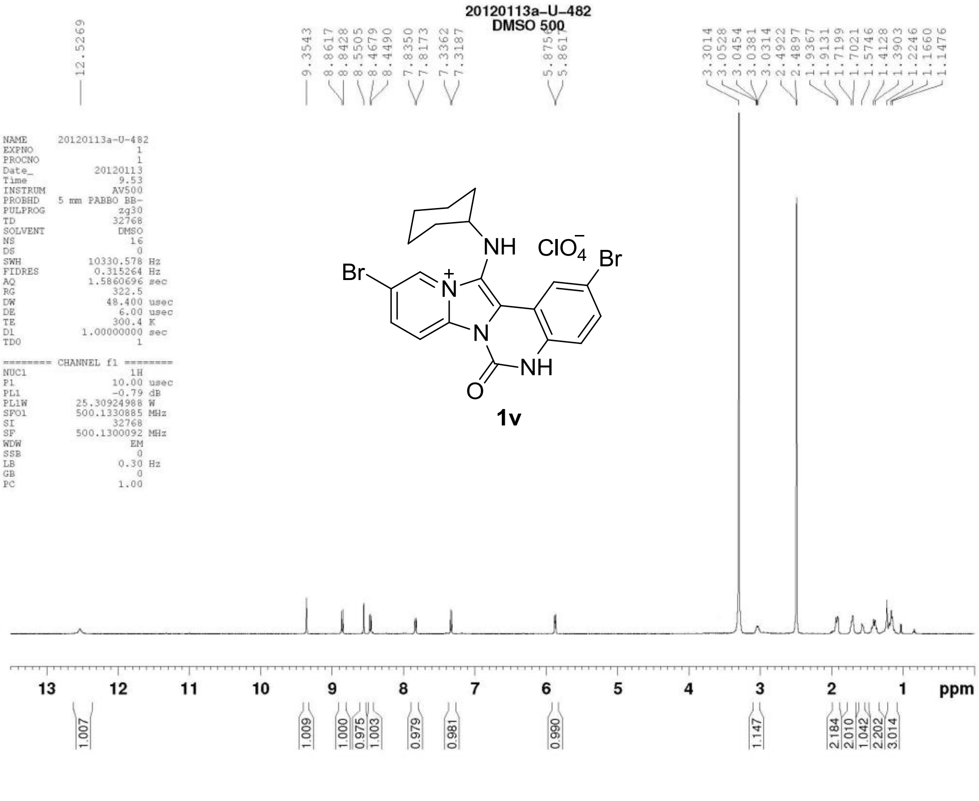

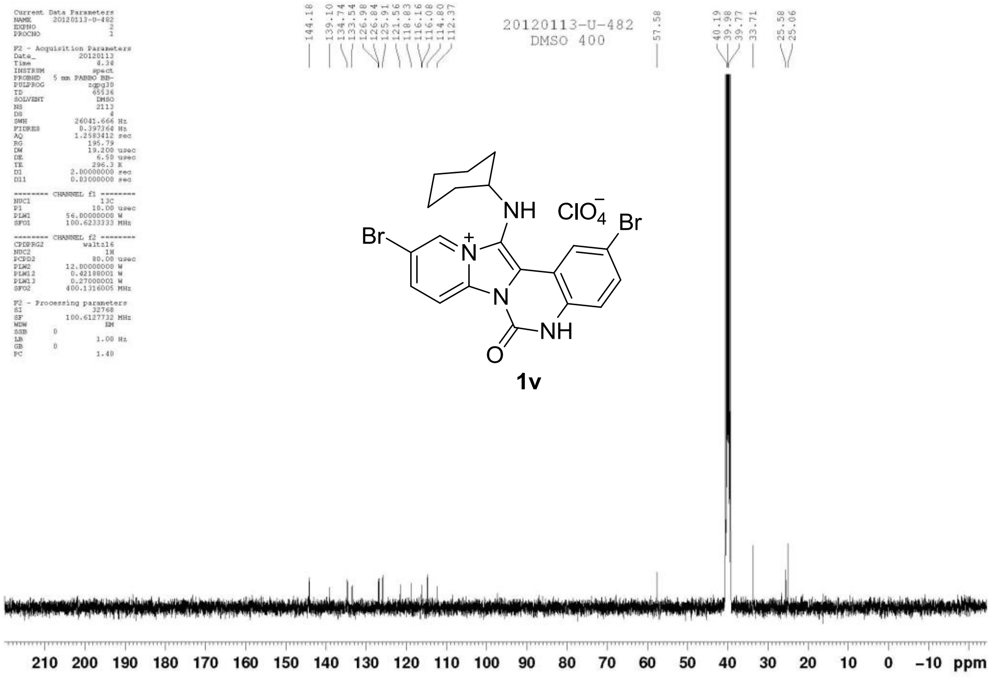

**1w**

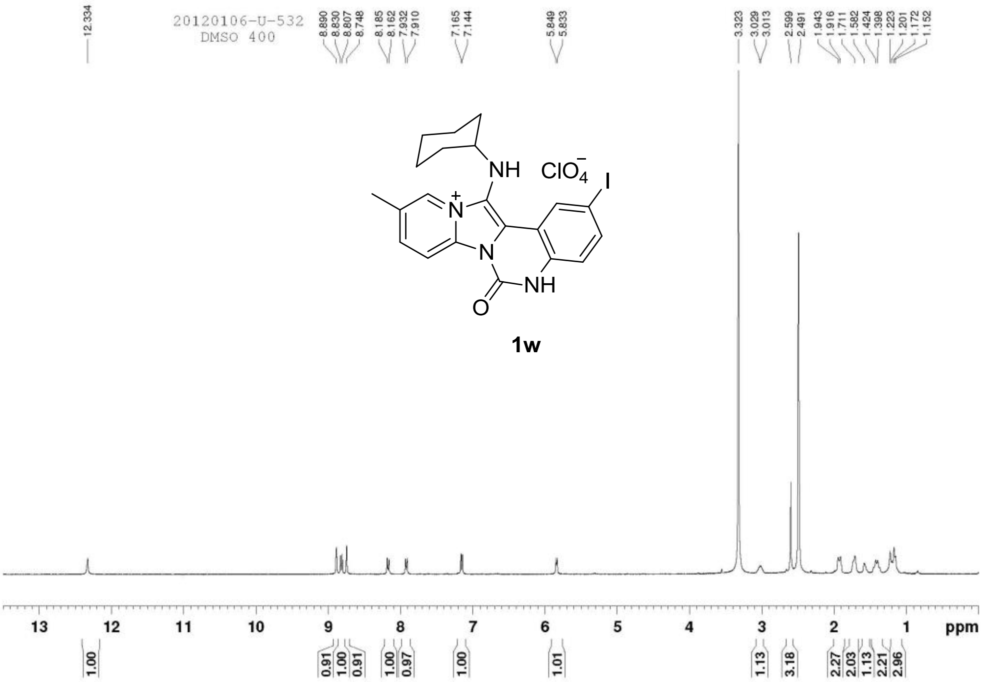

20120106-U-532
DMSO 400
12.334
8.890 8.830 8.807 8.748 8.185 8.162 7.932 7.910
7.165 7.144
5.849 5.833
3.323 3.029 3.013 2.599 2.491 1.943 1.916 1.711 1.582 1.424 1.398 1.223 1.201 1.172 1.152
NH
$ClO_4^-$
I
N
NH
O
1w
13 12 11 10 9 8 7 6 5 4 3 2 1 ppm
1.00 0.91 1.00 0.91 1.00 0.97 1.00 1.01 1.13 3.18 2.27 2.03 1.13 2.21 2.96


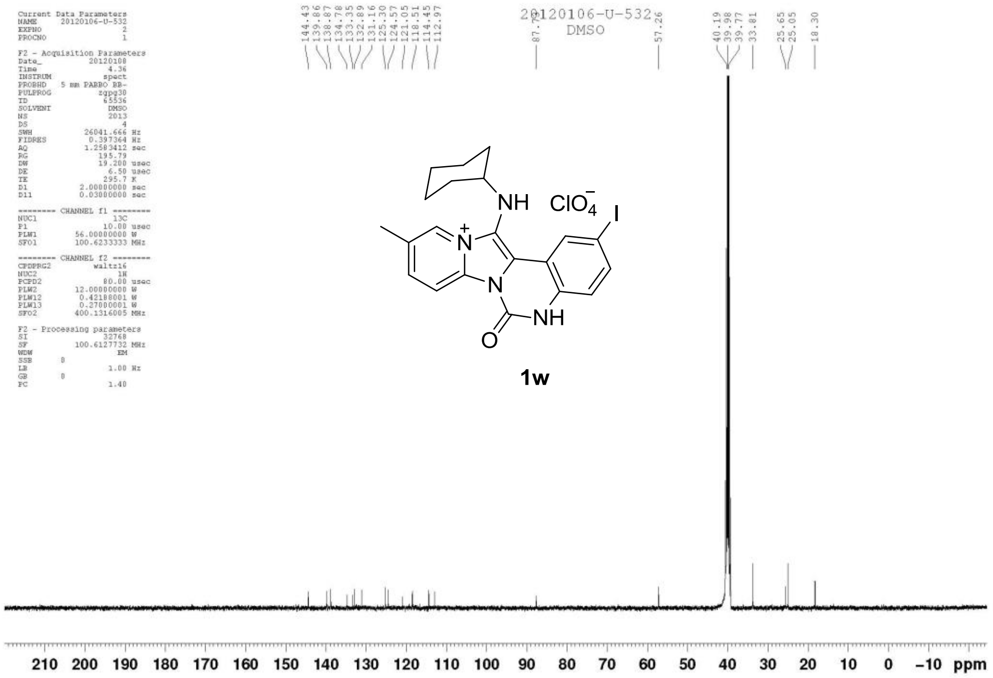

20120106-U-532
DMSO
144.43 139.86 138.87 134.78 133.35 132.89 131.16 125.30 124.57 121.05 118.51 114.45 112.97
57.26
40.19 39.98 39.77 33.81 25.65 25.05 18.30
NH
$ClO_4^-$
I
N
NH
O
1w
210 200 190 180 170 160 150 140 130 120 110 100 90 80 70 60 50 40 30 20 10 0 −10 ppm

**1x**

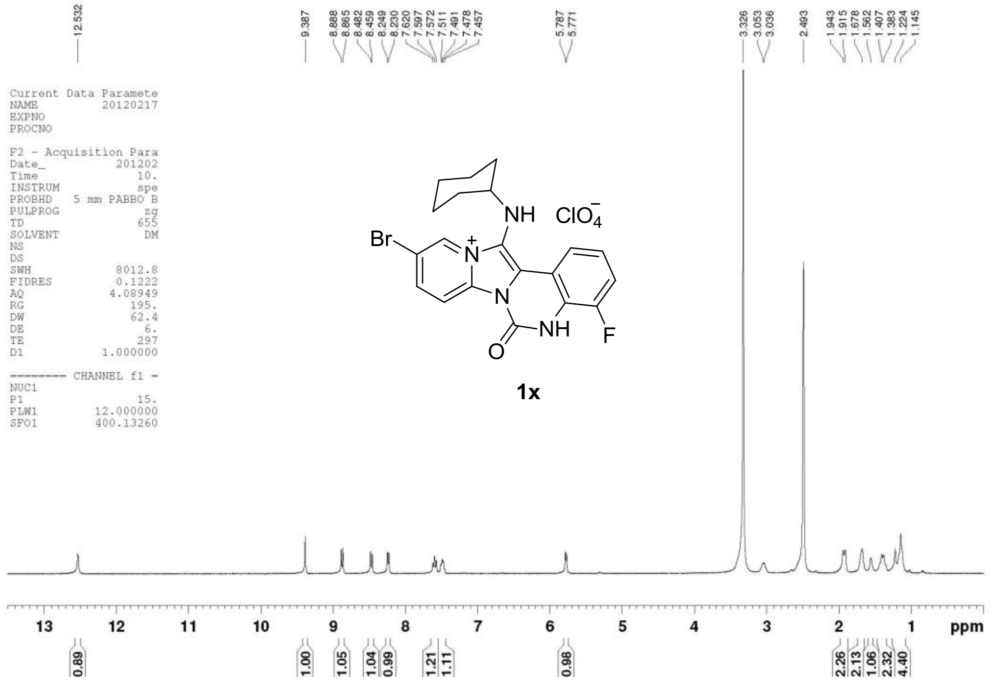

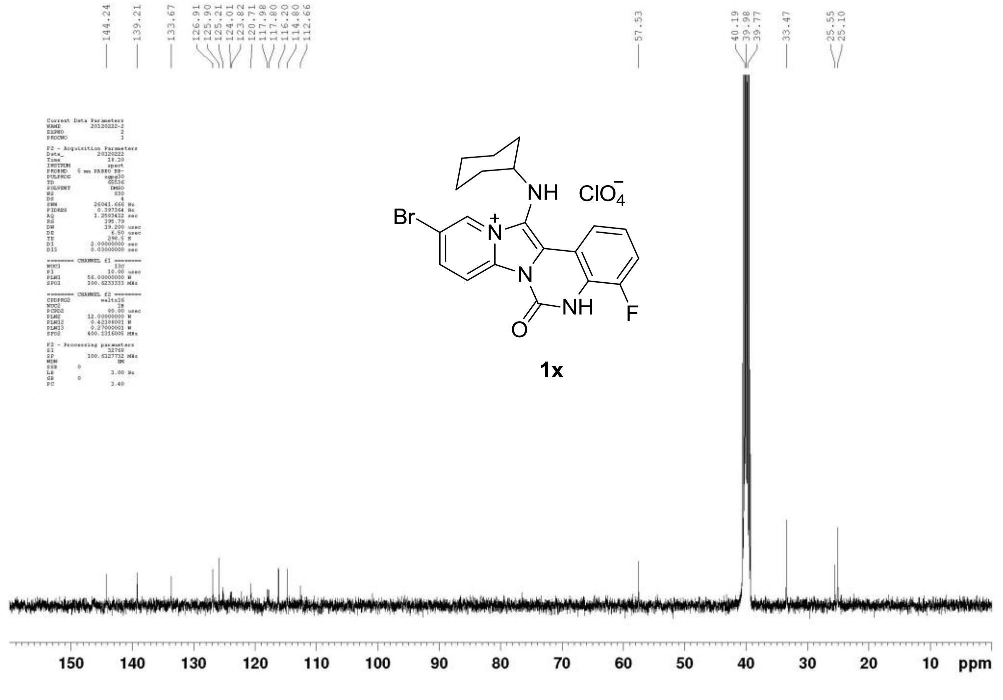

**1y**

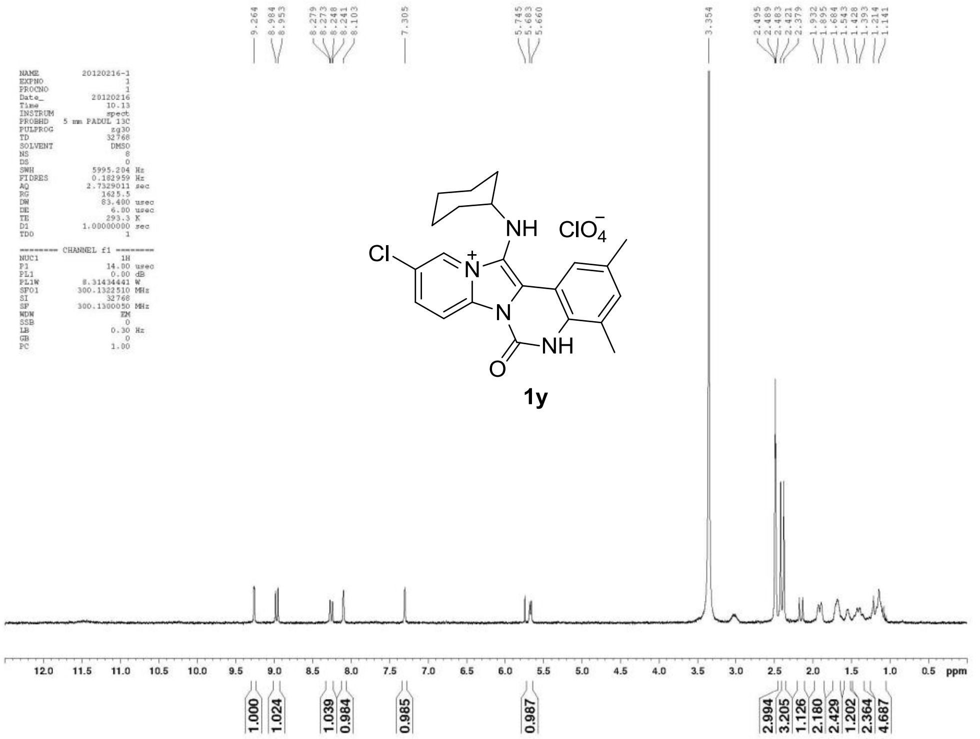

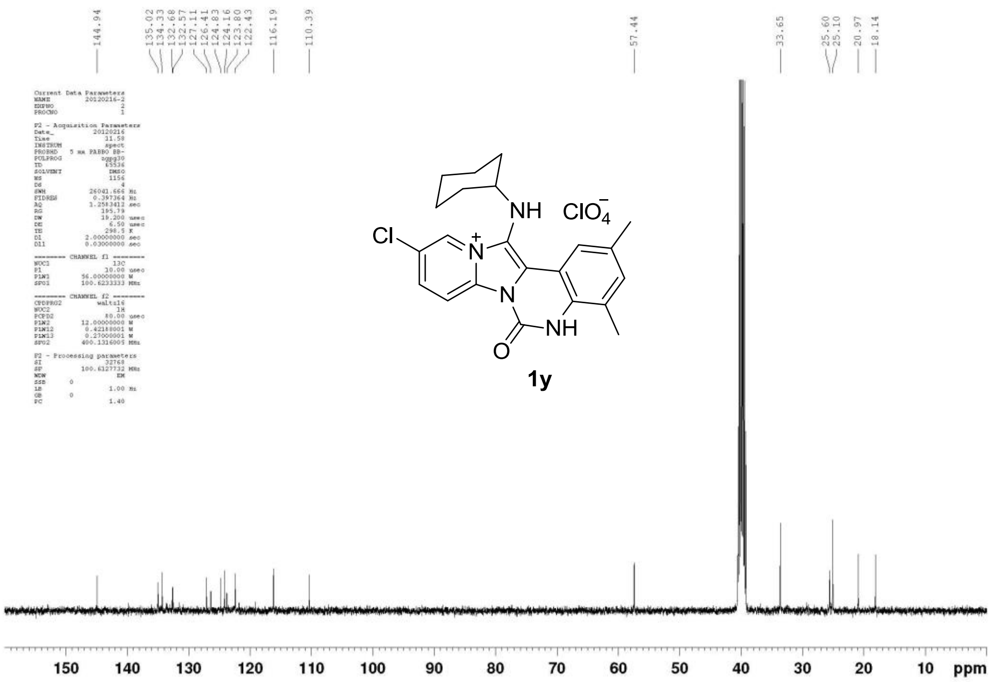


1y

# X-ray data for compound 1o

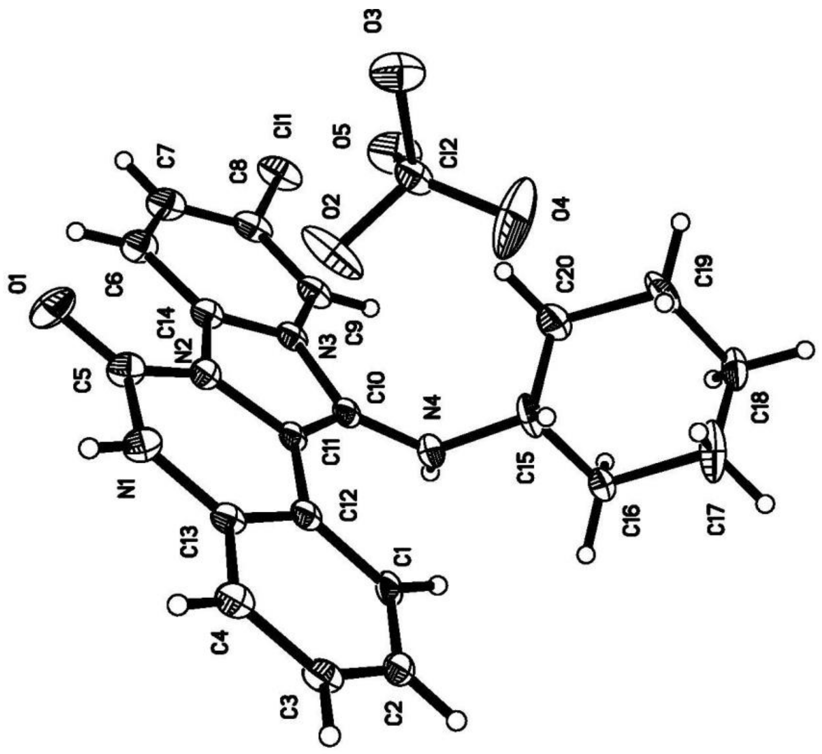


ORTEP structure for compound **1o**

**Table 1**. Crystal data and structure refinement for compound **1o**.

| | | |
|---|---|---|
| Identification code | 03071a | |
| Empirical formula | C20 H20 Cl2 N4 O5 | |
| Formula weight | 467.30 | |
| Temperature | 150(2) K | |
| Wavelength | 1.54184 Å | |
| Crystal system | Monoclinic | |
| Space group | P 21/c | |
| Unit cell dimensions | a = 9.9958(2) Å | $\alpha$= 90°. |
| | b = 15.0623(3) Å | $\beta$= 119.167(2)°. |
| | c = 15.2761(4) Å | $\gamma$ = 90°. |
| Volume | 2008.34(8) Å$^3$ | |
| Z | 4 | |
| Density (calculated) | 1.545 Mg/m$^3$ | |
| Absorption coefficient | 3.287 mm$^{-1}$ | |
| F(000) | 968 | |
| Crystal size | 0.38 x 0.34 x 0.25 mm$^3$ | |
| Theta range for data collection | 4.43 to 66.86°. | |
| Index ranges | -11<=h<=11, -14<=k<=17, -18<=l<=18 | |
| Reflections collected | 10354 | |
| Independent reflections | 3542 [R(int) = 0.0349] | |
| Completeness to theta = 66.86° | 99.6 % | |
| Absorption correction | Semi-empirical from equivalents | |
| Max. and min. transmission | 0.4937 and 0.3681 | |
| Refinement method | Full-matrix least-squares on F$^2$ | |
| Data / restraints / parameters | 3542 / 24 / 366 | |
| Goodness-of-fit on F$^2$ | 1.095 | |
| Final R indices [I>2sigma(I)] | R1 = 0.0535, wR2 = 0.1350 | |

| | |
| --- | --- |
| R indices (all data) | R1 = 0.0616, wR2 = 0.1439 |
| Largest diff. peak and hole | 0.346 and -0.858 e.Å$^{-3}$ |

**Table 2**. Atomic coordinates ( x $10^4$) and equivalent isotropic displacement parameters ($Å^2$x $10^3$) for compound **1o**.

U(eq) is defined as one third of the trace of the orthogonalized $U^{ij}$ tensor.

| | x | y | z | U(eq) |
|---|---|---|---|---|
| C(1) | 2496(3) | 10243(2) | 3302(2) | 32(1) |
| C(2) | 1349(3) | 10548(2) | 3488(2) | 36(1) |
| C(3) | 1150(3) | 10162(2) | 4237(2) | 35(1) |
| C(4) | 2082(3) | 9481(2) | 4818(2) | 32(1) |
| C(5) | 5412(3) | 8140(2) | 5161(2) | 32(1) |
| C(6) | 8023(3) | 7772(2) | 4638(2) | 37(1) |
| C(7) | 8947(3) | 7710(2) | 4208(2) | 40(1) |
| C(8) | 8624(3) | 8197(2) | 3346(2) | 36(1) |
| C(9) | 7418(3) | 8771(2) | 2929(2) | 31(1) |
| C(10) | 5204(3) | 9391(2) | 3079(2) | 27(1) |
| C(11) | 4690(3) | 9203(2) | 3737(2) | 25(1) |
| C(12) | 3452(3) | 9551(2) | 3875(2) | 27(1) |
| C(13) | 3254(3) | 9173(2) | 4639(2) | 27(1) |
| C(14) | 6757(3) | 8335(2) | 4174(2) | 28(1) |
| C(15) | 3453(3) | 9754(2) | 1300(2) | 37(1) |
| C(16) | 3218(13) | 10672(5) | 657(4) | 52(3) |
| C(17) | 1349(10) | 10290(7) | -336(5) | 61(2) |
| C(18) | 1947(13) | 9871(8) | -909(9) | 43(3) |
| C(19) | 2333(14) | 8940(5) | -272(5) | 52(2) |
| C(20) | 3987(16) | 9194(9) | 737(11) | 44(3) |
| C(15B) | 3453(3) | 9754(2) | 1300(2) | 37(1) |
| C(16B) | 2551(11) | 10483(6) | 753(6) | 39(2) |
| C(17B) | 1916(14) | 10566(7) | -422(6) | 49(3) |

| | | | | |
|---|---|---|---|---|
| C(18B) | 2252(17) | 9620(9) | -859(12) | 35(3) |
| C(19B) | 2963(13) | 8914(11) | -311(9) | 64(4) |
| C(20B) | 3830(20) | 9007(10) | 797(14) | 50(5) |
| Cl(1) | 9776(1) | 8073(1) | 2807(1) | 47(1) |
| N(1) | 4211(3) | 8495(2) | 5223(2) | 31(1) |
| N(2) | 5631(2) | 8553(1) | 4400(2) | 26(1) |
| N(3) | 6496(2) | 8830(1) | 3364(2) | 26(1) |
| N(4) | 4695(3) | 9980(2) | 2310(2) | 35(1) |
| O(1) | 6240(2) | 7555(1) | 5675(2) | 46(1) |
| Cl(2) | 2792(1) | 7038(1) | 2155(1) | 33(1) |
| O(2) | 3053(4) | 7401(3) | 3097(3) | 72(1) |
| O(3) | 2480(5) | 6082(3) | 2161(4) | 50(1) |
| O(4) | 1580(5) | 7456(4) | 1388(4) | 106(2) |
| O(5) | 4181(3) | 7094(2) | 2121(3) | 49(1) |
| Cl(2B) | 2792(1) | 7038(1) | 2155(1) | 33(1) |
| O(2B) | 4164(18) | 7195(10) | 2911(11) | 78(5) |
| O(3B) | 2014(19) | 6292(10) | 1917(13) | 39(4) |
| O(4B) | 1700(20) | 7652(12) | 2041(18) | 93(7) |
| O(5B) | 2822(17) | 7331(7) | 1133(9) | 58(4) |

**Table 3**. Bond lengths [Å] and angles [°] for compound **1o**.

| | |
|---|---|
| C(1)-C(2) | 1.387(4) |
| C(1)-C(12) | 1.397(4) |
| C(1)-H(1) | 0.9500 |
| C(2)-C(3) | 1.382(4) |
| C(2)-H(2) | 0.9500 |
| C(3)-C(4) | 1.379(4) |
| C(3)-H(3) | 0.9500 |
| C(4)-C(13) | 1.404(3) |
| C(4)-H(4) | 0.9500 |
| C(5)-O(1) | 1.203(3) |
| C(5)-N(1) | 1.358(3) |
| C(5)-N(2) | 1.426(3) |
| C(6)-C(7) | 1.373(4) |
| C(6)-C(14) | 1.395(4) |
| C(6)-H(6) | 0.9500 |
| C(7)-C(8) | 1.400(5) |
| C(7)-H(7) | 0.9500 |
| C(8)-C(9) | 1.363(4) |
| C(8)-Cl(1) | 1.723(3) |
| C(9)-N(3) | 1.377(3) |
| C(9)-H(9) | 0.9500 |
| C(10)-N(4) | 1.359(4) |
| C(10)-C(11) | 1.361(4) |
| C(10)-N(3) | 1.423(3) |
| C(11)-N(2) | 1.394(3) |
| C(11)-C(12) | 1.450(3) |
| C(12)-C(13) | 1.397(4) |

| | |
|---|---|
| C(13)-N(1) | 1.387(4) |
| C(14)-N(3) | 1.357(4) |
| C(14)-N(2) | 1.368(3) |
| C(15)-N(4) | 1.473(3) |
| C(15)-C(20) | 1.479(14) |
| C(15)-C(16) | 1.645(9) |
| C(15)-H(15A) | 1.0000 |
| C(16)-C(17) | 1.833(14) |
| C(16)-H(16A) | 0.9900 |
| C(16)-H(16B) | 0.9900 |
| C(17)-C(18) | 1.425(15) |
| C(17)-H(17A) | 0.9900 |
| C(17)-H(17B) | 0.9900 |
| C(18)-C(19) | 1.641(15) |
| C(18)-H(18A) | 0.9900 |
| C(18)-H(18B) | 0.9900 |
| C(19)-C(20) | 1.664(17) |
| C(19)-H(19A) | 0.9900 |
| C(19)-H(19B) | 0.9900 |
| C(20)-H(20A) | 0.9900 |
| C(20)-H(20B) | 0.9900 |
| C(16B)-C(17B) | 1.591(12) |
| C(16B)-H(16C) | 0.9900 |
| C(16B)-H(16D) | 0.9900 |
| C(17B)-C(18B) | 1.675(16) |
| C(17B)-H(17C) | 0.9900 |
| C(17B)-H(17D) | 0.9900 |
| C(18B)-C(19B) | 1.326(19) |
| C(18B)-H(18C) | 0.9900 |

| | |
|---|---|
| C(18B)-H(18D) | 0.9900 |
| C(19B)-C(20B) | 1.48(2) |
| C(19B)-H(19C) | 0.9900 |
| C(19B)-H(19D) | 0.9900 |
| C(20B)-H(20C) | 0.9900 |
| C(20B)-H(20D) | 0.9900 |
| N(1)-H(1A) | 0.84(3) |
| N(4)-H(4A) | 0.7692 |
| Cl(2)-O(4) | 1.362(4) |
| Cl(2)-O(5) | 1.417(2) |
| Cl(2)-O(2) | 1.439(3) |
| Cl(2)-O(3) | 1.474(4) |

| | |
|---|---|
| C(2)-C(1)-C(12) | 120.0(3) |
| C(2)-C(1)-H(1) | 120.0 |
| C(12)-C(1)-H(1) | 120.0 |
| C(3)-C(2)-C(1) | 119.9(3) |
| C(3)-C(2)-H(2) | 120.1 |
| C(1)-C(2)-H(2) | 120.1 |
| C(4)-C(3)-C(2) | 121.4(2) |
| C(4)-C(3)-H(3) | 119.3 |
| C(2)-C(3)-H(3) | 119.3 |
| C(3)-C(4)-C(13) | 119.1(3) |
| C(3)-C(4)-H(4) | 120.5 |
| C(13)-C(4)-H(4) | 120.5 |
| O(1)-C(5)-N(1) | 126.4(3) |
| O(1)-C(5)-N(2) | 121.4(2) |
| N(1)-C(5)-N(2) | 112.2(2) |
| C(7)-C(6)-C(14) | 117.0(3) |

| | |
|---|---|
| C(7)-C(6)-H(6) | 121.5 |
| C(14)-C(6)-H(6) | 121.5 |
| C(6)-C(7)-C(8) | 120.9(3) |
| C(6)-C(7)-H(7) | 119.6 |
| C(8)-C(7)-H(7) | 119.6 |
| C(9)-C(8)-C(7) | 121.3(2) |
| C(9)-C(8)-Cl(1) | 119.3(2) |
| C(7)-C(8)-Cl(1) | 119.4(2) |
| C(8)-C(9)-N(3) | 117.5(3) |
| C(8)-C(9)-H(9) | 121.2 |
| N(3)-C(9)-H(9) | 121.2 |
| N(4)-C(10)-C(11) | 131.1(2) |
| N(4)-C(10)-N(3) | 123.6(2) |
| C(11)-C(10)-N(3) | 105.3(2) |
| C(10)-C(11)-N(2) | 108.6(2) |
| C(10)-C(11)-C(12) | 133.4(2) |
| N(2)-C(11)-C(12) | 118.0(2) |
| C(1)-C(12)-C(13) | 119.7(2) |
| C(1)-C(12)-C(11) | 123.4(2) |
| C(13)-C(12)-C(11) | 116.9(2) |
| N(1)-C(13)-C(12) | 120.2(2) |
| N(1)-C(13)-C(4) | 119.8(2) |
| C(12)-C(13)-C(4) | 120.0(2) |
| N(3)-C(14)-N(2) | 106.4(2) |
| N(3)-C(14)-C(6) | 121.4(2) |
| N(2)-C(14)-C(6) | 132.2(3) |
| N(4)-C(15)-C(20) | 112.5(6) |
| N(4)-C(15)-C(16) | 103.5(3) |
| C(20)-C(15)-C(16) | 96.9(7) |

| | |
|---|---|
| N(4)-C(15)-H(15A) | 114.1 |
| C(20)-C(15)-H(15A) | 114.1 |
| C(16)-C(15)-H(15A) | 114.1 |
| C(15)-C(16)-C(17) | 91.3(7) |
| C(15)-C(16)-H(16A) | 113.4 |
| C(17)-C(16)-H(16A) | 113.4 |
| C(15)-C(16)-H(16B) | 113.4 |
| C(17)-C(16)-H(16B) | 113.4 |
| H(16A)-C(16)-H(16B) | 110.7 |
| C(18)-C(17)-C(16) | 94.8(8) |
| C(18)-C(17)-H(17A) | 112.8 |
| C(16)-C(17)-H(17A) | 112.8 |
| C(18)-C(17)-H(17B) | 112.8 |
| C(16)-C(17)-H(17B) | 112.8 |
| H(17A)-C(17)-H(17B) | 110.2 |
| C(17)-C(18)-C(19) | 93.5(10) |
| C(17)-C(18)-H(18A) | 113.0 |
| C(19)-C(18)-H(18A) | 113.0 |
| C(17)-C(18)-H(18B) | 113.0 |
| C(19)-C(18)-H(18B) | 113.0 |
| H(18A)-C(18)-H(18B) | 110.4 |
| C(18)-C(19)-C(20) | 101.1(8) |
| C(18)-C(19)-H(19A) | 111.5 |
| C(20)-C(19)-H(19A) | 111.5 |
| C(18)-C(19)-H(19B) | 111.5 |
| C(20)-C(19)-H(19B) | 111.5 |
| H(19A)-C(19)-H(19B) | 109.4 |
| C(15)-C(20)-C(19) | 101.1(9) |
| C(15)-C(20)-H(20A) | 111.6 |

| | |
|---|---|
| C(19)-C(20)-H(20A) | 111.6 |
| C(15)-C(20)-H(20B) | 111.6 |
| C(19)-C(20)-H(20B) | 111.6 |
| H(20A)-C(20)-H(20B) | 109.4 |
| C(17B)-C(16B)-H(16C) | 107.0 |
| C(17B)-C(16B)-H(16D) | 107.0 |
| H(16C)-C(16B)-H(16D) | 106.7 |
| C(16B)-C(17B)-C(18B) | 109.6(9) |
| C(16B)-C(17B)-H(17C) | 109.7 |
| C(18B)-C(17B)-H(17C) | 109.7 |
| C(16B)-C(17B)-H(17D) | 109.7 |
| C(18B)-C(17B)-H(17D) | 109.7 |
| H(17C)-C(17B)-H(17D) | 108.2 |
| C(19B)-C(18B)-C(17B) | 125.4(13) |
| C(19B)-C(18B)-H(18C) | 106.0 |
| C(17B)-C(18B)-H(18C) | 106.0 |
| C(19B)-C(18B)-H(18D) | 106.0 |
| C(17B)-C(18B)-H(18D) | 106.0 |
| H(18C)-C(18B)-H(18D) | 106.3 |
| C(18B)-C(19B)-C(20B) | 118.8(13) |
| C(18B)-C(19B)-H(19C) | 107.6 |
| C(20B)-C(19B)-H(19C) | 107.6 |
| C(18B)-C(19B)-H(19D) | 107.6 |
| C(20B)-C(19B)-H(19D) | 107.6 |
| H(19C)-C(19B)-H(19D) | 107.1 |
| C(19B)-C(20B)-H(20C) | 107.4 |
| C(19B)-C(20B)-H(20D) | 107.4 |
| H(20C)-C(20B)-H(20D) | 106.9 |
| C(5)-N(1)-C(13) | 127.0(2) |

| | |
|---|---|
| C(5)-N(1)-H(1A) | 113(2) |
| C(13)-N(1)-H(1A) | 120(2) |
| C(14)-N(2)-C(11) | 109.4(2) |
| C(14)-N(2)-C(5) | 125.0(2) |
| C(11)-N(2)-C(5) | 125.6(2) |
| C(14)-N(3)-C(9) | 121.9(2) |
| C(14)-N(3)-C(10) | 110.3(2) |
| C(9)-N(3)-C(10) | 127.8(2) |
| C(10)-N(4)-C(15) | 121.6(2) |
| C(10)-N(4)-H(4A) | 120.6 |
| C(15)-N(4)-H(4A) | 112.4 |
| O(4)-Cl(2)-O(5) | 114.7(3) |
| O(4)-Cl(2)-O(2) | 109.6(4) |
| O(5)-Cl(2)-O(2) | 108.07(19) |
| O(4)-Cl(2)-O(3) | 110.8(3) |
| O(5)-Cl(2)-O(3) | 105.9(2) |
| O(2)-Cl(2)-O(3) | 107.5(3) |

Symmetry transformations used to generate equivalent atoms:

**Table 4**. Anisotropic displacement parameters ($Å^2$x $10^3$) for compound **1o**.

The anisotropic displacement factor exponent takes the form: $-2\pi^2$[ $h^2$ $a^{*2}U^{11}$ + ... + 2 h k a* b* $U^{12}$ ]

| | $U^{11}$ | $U^{22}$ | $U^{33}$ | $U^{23}$ | $U^{13}$ | $U^{12}$ |
|---|---|---|---|---|---|---|
| C(1) | 18(1) | 50(2) | 20(1) | -8(1) | 1(1) | 7(1) |
| C(2) | 18(1) | 49(2) | 28(1) | -10(1) | 1(1) | 11(1) |
| C(3) | 16(1) | 47(2) | 38(2) | -17(1) | 11(1) | 1(1) |
| C(4) | 16(1) | 42(2) | 35(1) | -12(1) | 11(1) | -2(1) |
| C(5) | 24(1) | 35(1) | 40(2) | -2(1) | 18(1) | 2(1) |
| C(6) | 21(1) | 42(2) | 44(2) | -8(1) | 13(1) | 6(1) |
| C(7) | 19(1) | 44(2) | 52(2) | -13(1) | 14(1) | 3(1) |
| C(8) | 17(1) | 45(2) | 47(2) | -22(1) | 16(1) | -8(1) |
| C(9) | 20(1) | 42(2) | 33(1) | -16(1) | 14(1) | -9(1) |
| C(10) | 15(1) | 35(1) | 23(1) | -10(1) | 3(1) | 0(1) |
| C(11) | 13(1) | 33(1) | 21(1) | -10(1) | 2(1) | 0(1) |
| C(12) | 12(1) | 38(1) | 25(1) | -13(1) | 3(1) | 1(1) |
| C(13) | 15(1) | 33(1) | 30(1) | -11(1) | 8(1) | -2(1) |
| C(14) | 14(1) | 34(1) | 31(1) | -10(1) | 8(1) | -1(1) |
| C(15) | 20(1) | 71(2) | 18(1) | -8(1) | 7(1) | -4(1) |
| C(16) | 71(6) | 46(4) | 20(3) | -4(2) | 7(3) | 16(4) |
| C(17) | 38(4) | 104(7) | 26(3) | 21(4) | 4(3) | -13(4) |
| C(18) | 39(5) | 54(6) | 22(3) | 5(4) | 4(3) | -2(4) |
| C(19) | 65(6) | 50(4) | 27(3) | -19(3) | 11(4) | -20(4) |
| C(20) | 53(4) | 47(5) | 32(4) | -6(4) | 20(3) | -1(4) |
| C(15B) | 20(1) | 71(2) | 18(1) | -8(1) | 7(1) | -4(1) |
| C(16B) | 29(4) | 61(5) | 21(3) | 11(3) | 6(3) | -10(3) |
| C(17B) | 53(6) | 57(6) | 24(4) | -3(4) | 9(4) | 4(4) |
| C(18B) | 35(5) | 43(5) | 23(4) | -4(4) | 11(4) | -2(4) |

| | | | | | | |
|---|---|---|---|---|---|---|
| C(19B) | 28(5) | 129(11) | 40(5) | -43(6) | 20(4) | -22(6) |
| C(20B) | 89(9) | 24(5) | 26(5) | -6(4) | 20(5) | -29(5) |
| Cl(1) | 27(1) | 59(1) | 64(1) | -28(1) | 30(1) | -10(1) |
| N(1) | 24(1) | 34(1) | 41(1) | 0(1) | 19(1) | 3(1) |
| N(2) | 14(1) | 34(1) | 27(1) | -5(1) | 7(1) | 2(1) |
| N(3) | 14(1) | 35(1) | 27(1) | -13(1) | 8(1) | -3(1) |
| N(4) | 31(1) | 39(1) | 24(1) | -5(1) | 5(1) | 0(1) |
| O(1) | 39(1) | 43(1) | 65(1) | 19(1) | 33(1) | 19(1) |
| Cl(2) | 19(1) | 42(1) | 36(1) | -15(1) | 13(1) | -6(1) |
| O(2) | 82(3) | 93(3) | 64(2) | -55(2) | 53(2) | -48(2) |
| O(3) | 38(2) | 39(2) | 79(3) | -11(2) | 34(2) | -11(2) |
| O(4) | 41(2) | 145(5) | 91(4) | 58(4) | -1(2) | 20(3) |
| O(5) | 37(2) | 54(2) | 78(2) | -11(2) | 46(2) | -9(1) |
| Cl(2B) | 19(1) | 42(1) | 36(1) | -15(1) | 13(1) | -6(1) |
| O(2B) | 72(6) | 67(6) | 68(6) | -8(4) | 12(4) | -10(4) |
| O(3B) | 39(6) | 28(5) | 45(5) | -5(4) | 17(4) | -6(4) |
| O(4B) | 113(15) | 87(11) | 120(16) | 24(11) | 89(14) | 66(10) |
| O(5B) | 113(12) | 39(6) | 60(8) | 6(5) | 72(8) | 3(6) |

**Table 5**. Hydrogen coordinates ( x $10^4$) and isotropic displacement parameters ($Å^2$x $10^{3}$) for compound **1o**.

| | x | y | z | U(eq) |
|---|---|---|---|---|
| H(1) | 2630 | 10505 | 2784 | 39 |
| H(2) | 702 | 11021 | 3102 | 43 |
| H(3) | 354 | 10370 | 4354 | 42 |
| H(4) | 1933 | 9223 | 5331 | 38 |
| H(6) | 8236 | 7447 | 5225 | 44 |
| H(7) | 9817 | 7331 | 4499 | 48 |
| H(9) | 7222 | 9118 | 2359 | 38 |
| H(15A) | 2499 | 9534 | 1285 | 45 |
| H(16A) | 3984 | 10749 | 430 | 62 |
| H(16B) | 3156 | 11212 | 1006 | 62 |
| H(17A) | 850 | 9866 | -90 | 73 |
| H(17B) | 644 | 10785 | -699 | 73 |
| H(18A) | 2873 | 10169 | -845 | 52 |
| H(18B) | 1174 | 9798 | -1624 | 52 |
| H(19A) | 2450 | 8439 | -648 | 63 |
| H(19B) | 1532 | 8794 | -92 | 63 |
| H(20A) | 4523 | 8656 | 1120 | 53 |
| H(20B) | 4671 | 9527 | 555 | 53 |
| H(16C) | 3157 | 11025 | 1065 | 47 |
| H(16D) | 1653 | 10501 | 863 | 47 |
| H(17C) | 2426 | 11067 | -561 | 58 |
| H(17D) | 801 | 10686 | -764 | 58 |
| H(18C) | 1240 | 9408 | -1386 | 42 |
| H(18D) | 2830 | 9792 | -1207 | 42 |

| | | | | |
|---|---|---|---|---|
| H(19C) | 3683 | 8697 | -534 | 77 |
| H(19D) | 2185 | 8444 | -471 | 77 |
| H(20C) | 4930 | 9064 | 992 | 60 |
| H(20D) | 3720 | 8443 | 1088 | 60 |
| H(1A) | 4130(40) | 8290(20) | 5710(30) | 38 |
| H(4A) | 5231 | 10341 | 2299 | 42 |

**Table 6**. Torsion angles [°] for compound **1o**.

| | |
|---|---|
| C(12)-C(1)-C(2)-C(3) | -0.4(4) |
| C(1)-C(2)-C(3)-C(4) | 0.8(4) |
| C(2)-C(3)-C(4)-C(13) | -0.2(4) |
| C(14)-C(6)-C(7)-C(8) | 0.4(4) |
| C(6)-C(7)-C(8)-C(9) | 2.4(4) |
| C(6)-C(7)-C(8)-Cl(1) | -177.9(2) |
| C(7)-C(8)-C(9)-N(3) | -2.4(4) |
| Cl(1)-C(8)-C(9)-N(3) | 177.97(18) |
| N(4)-C(10)-C(11)-N(2) | -178.9(2) |
| N(3)-C(10)-C(11)-N(2) | 0.1(3) |
| N(4)-C(10)-C(11)-C(12) | -0.7(5) |
| N(3)-C(10)-C(11)-C(12) | 178.3(2) |
| C(2)-C(1)-C(12)-C(13) | -0.4(4) |
| C(2)-C(1)-C(12)-C(11) | -179.1(2) |
| C(10)-C(11)-C(12)-C(1) | -2.3(4) |
| N(2)-C(11)-C(12)-C(1) | 175.7(2) |
| C(10)-C(11)-C(12)-C(13) | 178.9(3) |
| N(2)-C(11)-C(12)-C(13) | -3.0(3) |
| C(1)-C(12)-C(13)-N(1) | -179.1(2) |
| C(11)-C(12)-C(13)-N(1) | -0.3(3) |
| C(1)-C(12)-C(13)-C(4) | 1.0(4) |
| C(11)-C(12)-C(13)-C(4) | 179.7(2) |
| C(3)-C(4)-C(13)-N(1) | 179.4(2) |
| C(3)-C(4)-C(13)-C(12) | -0.7(4) |
| C(7)-C(6)-C(14)-N(3) | -3.2(4) |
| C(7)-C(6)-C(14)-N(2) | -179.5(3) |
| N(4)-C(15)-C(16)-C(17) | -163.7(4) |

| | |
|---|---|
| C(20)-C(15)-C(16)-C(17) | 81.1(8) |
| C(15)-C(16)-C(17)-C(18) | -90.7(9) |
| C(16)-C(17)-C(18)-C(19) | 85.5(8) |
| C(17)-C(18)-C(19)-C(20) | -80.8(10) |
| N(4)-C(15)-C(20)-C(19) | 172.4(5) |
| C(16)-C(15)-C(20)-C(19) | -79.9(9) |
| C(18)-C(19)-C(20)-C(15) | 76.4(10) |
| C(16B)-C(17B)-C(18B)-C(19B) | -1(2) |
| C(17B)-C(18B)-C(19B)-C(20B) | -14(2) |
| O(1)-C(5)-N(1)-C(13) | 179.6(3) |
| N(2)-C(5)-N(1)-C(13) | 0.4(4) |
| C(12)-C(13)-N(1)-C(5) | 1.7(4) |
| C(4)-C(13)-N(1)-C(5) | -178.3(3) |
| N(3)-C(14)-N(2)-C(11) | -1.3(3) |
| C(6)-C(14)-N(2)-C(11) | 175.4(3) |
| N(3)-C(14)-N(2)-C(5) | 175.4(2) |
| C(6)-C(14)-N(2)-C(5) | -7.9(4) |
| C(10)-C(11)-N(2)-C(14) | 0.8(3) |
| C(12)-C(11)-N(2)-C(14) | -177.7(2) |
| C(10)-C(11)-N(2)-C(5) | -175.9(2) |
| C(12)-C(11)-N(2)-C(5) | 5.6(3) |
| O(1)-C(5)-N(2)-C(14) | 0.4(4) |
| N(1)-C(5)-N(2)-C(14) | 179.7(2) |
| O(1)-C(5)-N(2)-C(11) | 176.6(3) |
| N(1)-C(5)-N(2)-C(11) | -4.1(4) |
| N(2)-C(14)-N(3)-C(9) | -179.6(2) |
| C(6)-C(14)-N(3)-C(9) | 3.3(4) |
| N(2)-C(14)-N(3)-C(10) | 1.4(3) |
| C(6)-C(14)-N(3)-C(10) | -175.8(2) |

| | |
|---|---|
| C(8)-C(9)-N(3)-C(14) | -0.4(3) |
| C(8)-C(9)-N(3)-C(10) | 178.5(2) |
| N(4)-C(10)-N(3)-C(14) | 178.1(2) |
| C(11)-C(10)-N(3)-C(14) | -0.9(3) |
| N(4)-C(10)-N(3)-C(9) | -0.9(4) |
| C(11)-C(10)-N(3)-C(9) | -179.9(2) |
| C(11)-C(10)-N(4)-C(15) | -85.6(3) |
| N(3)-C(10)-N(4)-C(15) | 95.6(3) |
| C(20)-C(15)-N(4)-C(10) | -78.4(7) |
| C(16)-C(15)-N(4)-C(10) | 178.2(4) |

Symmetry transformations used to generate equivalent atoms:

**Table 7**. Hydrogen bonds for compound **1o** [Å and °].

| D-H...A | d(D-H) | d(H...A) | d(D...A) | <(DHA) |
|---|---|---|---|---|
| N(4)-H(4A)...O(3)#1 | 0.77 | 2.31 | 3.026(5) | 156.1 |
| N(1)-H(1A)...O(5)#2 | 0.84(3) | 2.22(4) | 3.047(4) | 171(3) |

Symmetry transformations used to generate equivalent atoms:

#1 -x+1,y+1/2,-z+1/2 #2 x,-y+3/2,z+1/2
